# Review: Recent Applications of Gene Editing in Fish Species and Aquatic Medicine

**DOI:** 10.3390/ani13071250

**Published:** 2023-04-04

**Authors:** Anikó Gutási, Sabine E. Hammer, Mansour El-Matbouli, Mona Saleh

**Affiliations:** 1 Department of Farm Animals and Veterinary Public Health, Division of Fish Health, University of Veterinary Medicine, 1210 Vienna, Austria; 2 Department of Pathobiology, Institute of Immunology, University of Veterinary Medicine, 1210 Vienna, Austria

**Keywords:** gene editing, gene silencing, aquatic animals, targeted modification, CRISPR/Cas9, TALENs, Zink finger nucleases, fish, crustaceans

## Abstract

**Simple Summary:**

The aquaculture industries are essential sectors of food production and global trade. Several novel approaches have established gene modification in different fish species over the last few years. These approaches show that gene editing tools, including the CRISPR/Cas9 technique, are very powerful and broadly used in aquaculture. The targeted and accurate modifications in the genome of different fish species and their pathogens bring radical improvement in different aquaculture sectors, including disease resistance, growth or reproduction. With these novel techniques presenting feasible molecular devices, the development of functional genomics and therapeutic applications in fish species and crustaceans can be enhanced. In summary, the creation of mutant animals in aquaculture through specific gene modification methods is the reality.

**Abstract:**

Gene editing and gene silencing techniques have the potential to revolutionize our knowledge of biology and diseases of fish and other aquatic animals. By using such techniques, it is feasible to change the phenotype and modify cells, tissues and organs of animals in order to cure abnormalities and dysfunctions in the organisms. Gene editing is currently experimental in wide fields of aquaculture, including growth, controlled reproduction, sterility and disease resistance. Zink finger nucleases, TALENs and CRISPR/Cas9 targeted cleavage of the DNA induce favorable changes to site-specific locations. Moreover, gene silencing can be used to inhibit the translation of RNA, namely, to regulate gene expression. This methodology is widely used by researchers to investigate genes involved in different disorders. It is a promising tool in biotechnology and in medicine for investigating gene function and diseases. The production of food fish has increased markedly, making fish and seafood globally more popular. Consequently, the incidence of associated problems and disease outbreaks has also increased. A greater investment in new technologies is therefore needed to overcome such problems in this industry. To put it concisely, the modification of genomic DNA and gene silencing can comprehensively influence aquatic animal medicine in the future. On the ethical side, these precise genetic modifications make it more complicated to recognize genetically modified organisms in nature and can cause several side effects through created mutations. The aim of this review is to summarize the current state of applications of gene modifications and genome editing in fish medicine.

## 1. Introduction

### 1.1. Fish Industry

Global fish production became the fastest growing food technology in the major food yield in the past decades. Aquaculture produces more fish biomass than the production of the whole beef biomass around the world, and more biomass than capture fisheries (included the amount of non-edible species) [1].

In the twenty-first century, the enlargement in aquaculture and fisheries production is notable [2]. The world aquaculture production increased quickly from 5 million to 63 million tons. In addition, capture fisheries’ production increased from 69 million to 93 million tons over the last three decades [3]. Food fish consumption grew around 1.4 percent yearly, from 9.0 kg in 1961 to 20.5 kg per capita in 2019 [2].

Global aquaculture production included 178 million tonnes of food fish in the year 2020. Farmed food fish produced 57.5 million tonnes of finfish, 17.7 million tonnes of molluscs, 11.2 million tonnes of crustaceans, 525,000 tonnes of other aquatic invertebrates and 537,000 tonnes of semi-aquatic species, including turtles and frogs, in 2020. Asia has been in first place for decades, with 91.6 percent of the global aquaculture production (animals and algae) in 2020. The most dominant producer of farmed food fish was China in 2020, and they have produced more farmed aquatic animals and algae than the rest of the world combined every year since 1991. The other major producing countries were Vietnam, Bangladesh, Egypt, Norway and Chile in 2005–2020.

In 2050, the Earth’s population will probably reach 10 billion. The food production sector needs to be more effective in utilizing productive resources. Fish can be globally advantageous in feeding and nutritional security among the poor and vulnerable society [2]. Fish is an excellent nutrition source; it has several positive values. It provides not only high-value protein, but it is also low in saturated fats, carbohydrates and cholesterol, and contains several vitamins, minerals and polyunsaturated omega-3 fatty acids [4]. The farming of fed aquatic animal species has outpaced the farming of unfed species. Both types of farming systems have expanded continuously, but the volume of fed species has grown faster than the non-fed species. It appears that the production of food fish has increased intensely, making fish and seafood globally more popular and reducing the price of fish. Therefore, greater investment is needed in new technologies in the industry [2,5]. Climate change presents the most serious challenge to a growing level of sustainable global aquaculture. Definitive climate-induced changes in physical and biological conditions may require us to modify management practices in the future [6]. Genetically modified aquatic animals are of great interest and could bring benefits for the quickly increasing aquaculture production to feed the growing human population globally and for curing inherited diseases in aquaculture [2]. 

### 1.2. Gene Editing

Gene engineering techniques have been employed by scientists and researchers to answer some combined questions in biology. In the late 1980s and early 1990s, attempts were made to precisely modify the complex genome [7]. Thanks to the developed techniques, it was possible to decipher the DNA structure, its replication, transcription and translation of genomics. Furthermore, new genetic engineering techniques have revolutionized the genetic manipulation, creating a significant impact on modern medicine, principally gene therapy [8,9]. Gene editing is a very effective technique in which the DNA or nucleotide sequences are inserted, deleted or replaced at a specific place in the genome of living organisms or cells using a specific set of engineered nucleases as molecular scissors. It is possible to induce precise, favorable changes: for example, fixing alleles at existing trait loci, or introducing alleles from different strains or species [9,10]. Although the process causes breaks in the gene, these can later be repaired via activation of a DNA repair mechanism, hence parts of the DNA can be positioned into site-specific regions in the genome of interest [11]. The phenotypic characteristics of an organism can also be modified by creating exact and special changes in the genome [9]. Thanks to the gene editing tools and technologies, diverse fields and departments have been covered, which can help researchers to develop new experiments, like veterinary medicine [11]. Genome engineering can be applied in the treatment of genetic diseases, different chronic health diseases and cancer, as well as in the management of diseases [9]. The most often used methods in gene editing, which are widely successful, utilize sequence-specific programmable nucleases [12,13]. These molecular scissors precisely cut the DNA at a special localisation [14].

**Figure 1:**
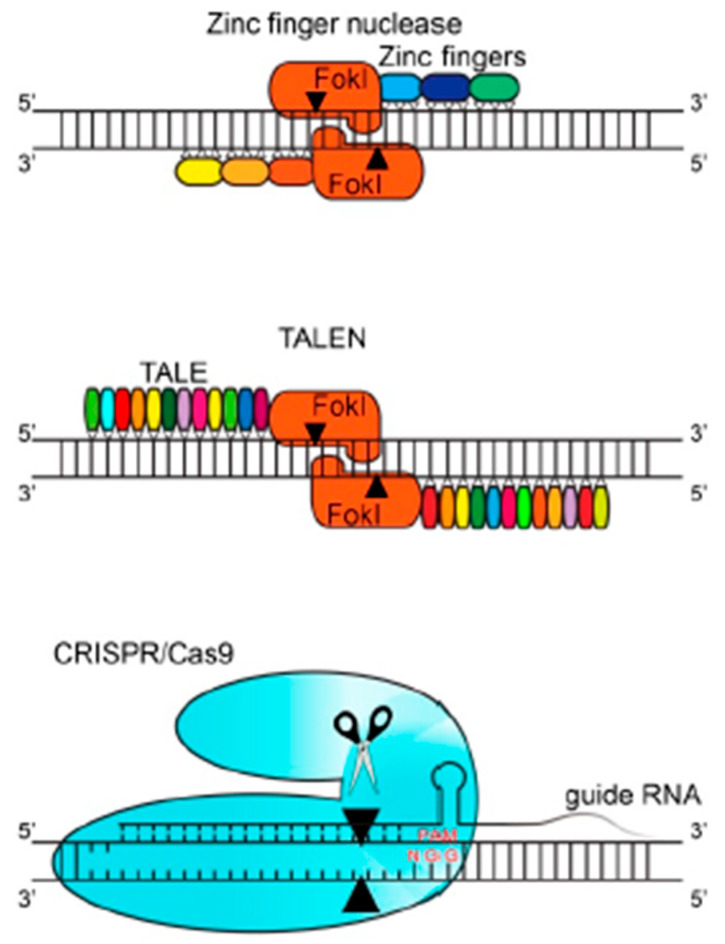
Overview of the diverse gene editing nucleases (ZFN, TALEN, CRISPR/Cas9). The black arrows illustrate the cleavage site of the DNA [15].

These specific nucleases include two different types (Figure 1). Zinc finger nucleases (ZFNs) and transcription activator-like effector nucleases (TALENs) are both protein-guided, whereas the two component CRISPR/Cas systems contain RNA-guided endonucleases (RGENs) [9,15] (Table 1).

All these methods can generate targeted double-strand DNA breaks (DSBs) in the DNA. The generated double-strand breaks can lead to a loss of large chromosomal regions, which may cause the most hazardous type of DNA damage. The two major types of the endogenous cellular DNA repair pathways are nonhomologous end-joining (NHEJ) and homologous recombinational (HR) repair or homology-directed repair HDR in eukaryotic cells [16,17,18,19].

**Figure 2:**
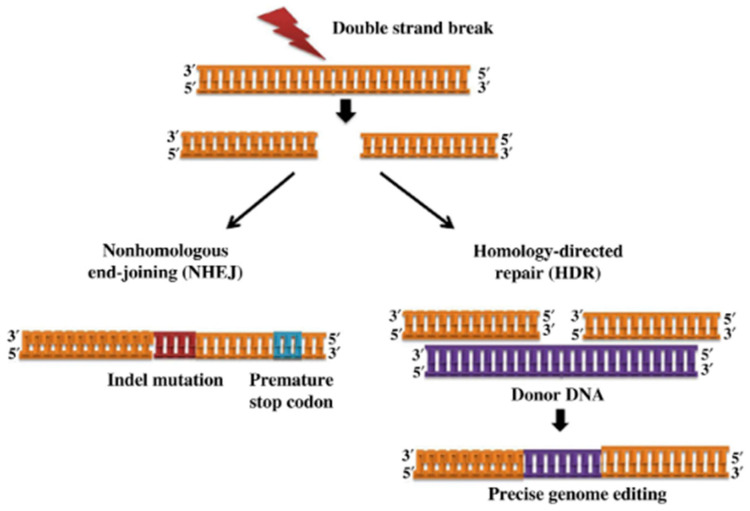
DNA double-strand breaks into genomic loci are repaired by various strategies, including nonhomologous end-joining (NHEJ) or homology-directed repair (HDR) pathway [25].

During the subpathway of NHEJ, the break ends are identified, resected, polymerized and ligated by proteins in flexible mode [19]. The break ends are directly ligated; a homologous template is not required for the repair (Figure 2). This method is an error-prone process, which often comes with imprecise repairs, such as the loss/gain of some nucleotides. Therefore, the result is variable, and deletions and insertions of nucleotide or nucleotide substitutions occurs in the broken region [9,20]. The mechanism comprises individual and sequential steps: (1) identification of DNA end, assembly and stabilization the NHEJ complex at the place of DNA double-strand break; (2) bridging of the DNA ends and support of break end stability; (3) processing of DNA end; (4) ligation of the DNA broken ends and dissolution of the NHEJ complex [21]. The homology-directed repair (HDR) mechanism can be exploited by the cells when there is homologous DNA as a template to restore DSBs [22]. Following the introduction of a DSB into the genome, proteins are enlisted to the exposed the ends of DNA to start repair of the break [23]. The result of this type of repair is precise and controlable and effectively useful to correctly edit genomic sequence, to induce specific deletions, insertions or designer mutations, as well as to insert an exogenous sequence [22]. It occurs low in post-mitotic and differentiated cells. The effectiveness of HDR is highly determined by the target locus of the genome and the template itself, as well as the cell type and stage of life [24]. 

#### 1.2.1. Zinc Finger Nucleases (ZFNs)

Zinc finger nucleases are artificially engineered hybrid proteins that are widely used as a potential gene editing tool [26]. The principle is that different zinc fingers identify different sets of nucleotide triplets. This hybrid protein consists of specific DNA-binding domains that fuse with the endonuclease Fok I, created to target specific genome sequences [26,27,28,29]. Zinc finger proteins (ZFPs) have a unique ability to recognize and bind to specific DNA sequences, and ZFN enzymes can cut the DNA in the targeted sequences [29]. In addition, ZFN can create a DNA double-strand break (DSB) at preselected sites [8]. The significant concern for ZFNs is off-target cleavage, contrary to many natural endonucleases [30]. ZFNs and ZFPs are classified in three major subtypes (C2H2, C4 and C6), in which C2H2 is, due to its simplicity, the most broadly used in engineered ZFNs [29,31]. The major advantage of this technique compared to standard gene therapy is the potential to conserve temporal and tissue-specific gene expression [8]. 

**Figure 3:**
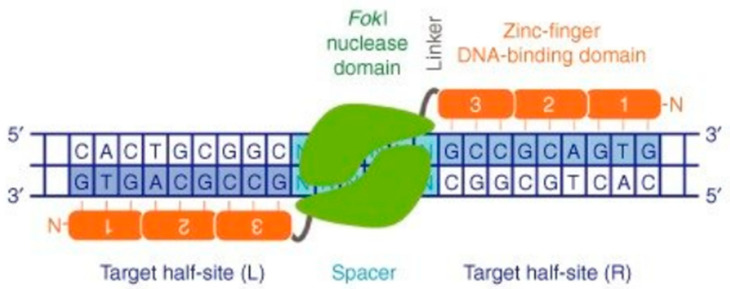
Zinc finger nuclease (ZFN)-mediated genome editing [8].

Figure 3 shows the architecture of ZFNs. The two monomer subunits of the multimerized ZFNs bind to the target locus of the DNA sequence. Each subunit contains three zinc-fingers, which identify nine base pairs within the full target site and the Fok I endonuclease domain. The two short linkers associate with two domains. After the dimerization of the two subunits, the nuclease is activated and cuts the DNA in the “spacer” sequence.

ZFN creates a double-strand break (DSB) and separates the two target half sites, (L) and (R) [8]. After the break occurs, the error-prone NHEJ process and the DNA repair takes place [9]. The ZFNs can individually contain between three and six zinc finger domains that each recognizes and binds between 9 and 18 base pairs at the target site [32]. The three zinc finger motifs monomer is the minimal requirement, and it was also reported that the strings with three to four zinc finger motifs have the highest binding ability [29]. In summary, the endonuclease activity together with special nucleotide sequence binding particularities of ZFs takes part in genome engineering via targeted DSB formation. Several successful endonuclease-mediated gene editing attempts have been applied in different species, thanks to the high conservation of the DNA-repair mechanism [33]. This application has been used to manipulate the genome, for example, of zebrafish [34] and of numerous human cells, including primary somatic cells [35] and embryonic stem cells [36]. 

#### 1.2.2. Transcriptional Activator-Like Effector Nucleases (TALENs)

**Figure 4:**
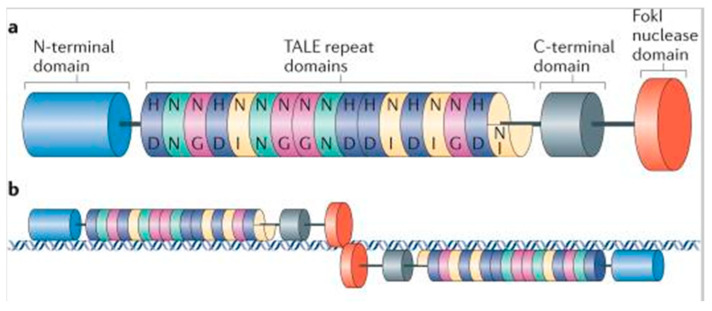
(**a**) Schematic diagram of a TALEN. (**b**) TALENs bind and cleave as dimers on a target DNA site and cause DSBs in the “spacer” sequence [37].

TALENs consist of special effector proteins, which contain the DNA-binding domain and Fok 1 nuclease domain. These domains work in pairs as dimers, binding to the opposite strand DNA and inducing DSB. TALENs are applied for genome editing and introducing targeted DSBs into specific DNA sites of interest, as an alternative to ZFNs [8,37]. These unique nucleases are secreted by the pathogenic bacteria Xanthomonas, which infect the cytoplasm of plant cells [38]. Each of these nuclease platforms has a central domain for the special DNA binding and distinct N- and C-termini architectures for localization and activation [37,39,40,41]. The DNA-binding domain comprises monomers with 10 to 30 repeats and each of them binds with one nucleotide of the target DNA sequence [12,41]. Besides, they comprise a non-specific FokI catalytic nuclease domain combined to a customizable DNA-binding domain. TALENs bind as dimers to the target sites in the nucleus with the FokI domains located at the c-termini and cleavage occurring in the “spacer” sequence [37,42]. Thereafter, the repair of DNA breaks, which occurs primarily in the same way as ZFNs, the error-prone NHEJ method [8]. TALENS can be very easily and rapidly designed (Figure 4).

Their high rates of cleavage activity and their relevant limitless targeting array make them appropriate for gene mutation purposes [37]. Taken together, the TALEN-based method requires engineering a pair of large repetitive sequence-encoding domains for site-specific DNA identification and cleavage in the genome [9]. With the use of TALEN, efficient introduction of targeted modifications has been achieved in numerous model organisms [37]. However, their highly repetitive sequences make long TALE repeats frequently inefficient, labor consuming and expensive to create. For this reason, there was demanded to develop new simpler, more rapid, robust, more efficient and cost-effective techniques for gene editing in the biomedical field [9]. 

#### 1.2.3. Clustered Regularly Interspaced Short Palindromic Repeats—Cas 9 System (CRISPR/Cas9)

**Figure 5:**
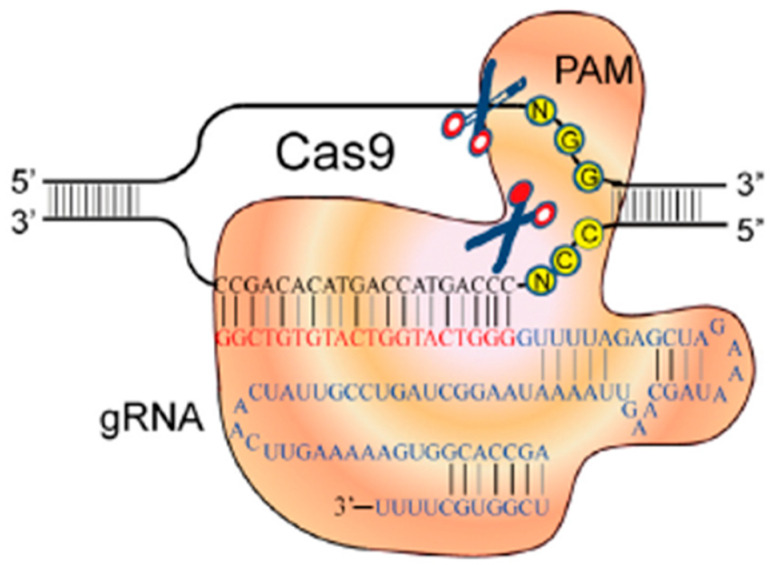
Genome editing with CRISPR/Cas9 [52].

The clustered regularly interspaced short palindromic repeats (CRISPR) gene engineering technique is one of the latest trends in the genome editing toolbox. This most recent gene editing method was discovered in 2012 [43]. It is a significant technical jump forward for biomedical applications and research, as well as one of the fastest to progress to the use for precise gene modification in different organisms [9,43,44]. It has various advantages over the above-mentioned processes (ZFNs—and TALENs—based). It is more effective, much simpler to accomplish and appropriate for high-performance and multiple gene editing in many living organisms and cell lines [9]. Due to its simplicity, speed and low cost to devise, it is widely adopted and is now the technology of choice [9,45,46]. It works in simple, as well as more complex cells [47]. This system was utilized to develop RNA-guided endonucleases that enable targeted genome editing [9]. Originally, it was naturally present in prokaryotic cells, namely in bacteria and archaea [48]. There are at least 11 diverse CRISPR/Cas systems, which have been categorized into three groups according to the attribute of the Cas protein: type Ⅰ, type Ⅱ and type Ⅲ. The type Ⅱ system uses only one Cas protein to identify and cleave targeted DNA sites, while different type Ⅰ and type Ⅲ systems expect a set of Cas proteins [49,50,51]. Because of the simplicity of the type Ⅱ system, which is also known as CRISPR/Cas9, it has been considered a potent programmable mechanism to specific modifications in the genome [9] (Figure 5).

The CRISPR/Cas9 system encompasses a Cas9 endonuclease and a modified single guide RNA (sgRNA/gRNA), which comprises a targeting CRISPR RNA (crRNA) and trans-activating crRNA (tracrRNA) [43]. Hence, the two essential components are the Cas9 protein and sgRNA [53]. The Cas9 nuclease is directed to its target sequence by a precisely designed guide RNA of about 20 base pairs [54]. Thus, one of the great advantages of this system is that it requires a simple change of 20 nucleotide sgRNA “spacer” sequences, which is easier to manipulate, and not the large repetitive complex design of DNA-binding arrays for each novel genomic target site, as in ZFN and TALEN systems [9,37]. Another important part is the protospacer-adjacent motif (PAM) of the target sequence, which binds with Cas9. PAM is a short, specific sequence (NGG trinucleotide sequence) following the target DNA sequence, presenting a downstream of the crRNA binding site. It is necessary for a Cas nuclease-mediated break. The cleavage of the DNA is carried out by the Cas9 enzyme at position 3–4 nucleotides upstream of PAM [9,43]. Summarily, the Cas9 endonuclease precisely cuts the target DNA sequence and introduces a DSB under the control of sgRNA. Accordingly, researchers can add or delete sequences of the genetic material or switch an existing segment with an altered sequence of the DNA to create modifications. A DBS can be repaired either via NHEJ or HDR [9,55]. This system provides several innovative opportunities in addition to applications for genome editing techniques of both in vivo and in vitro systems [15]. 

#### 1.2.4. Gene Silencing

**Figure 6:**
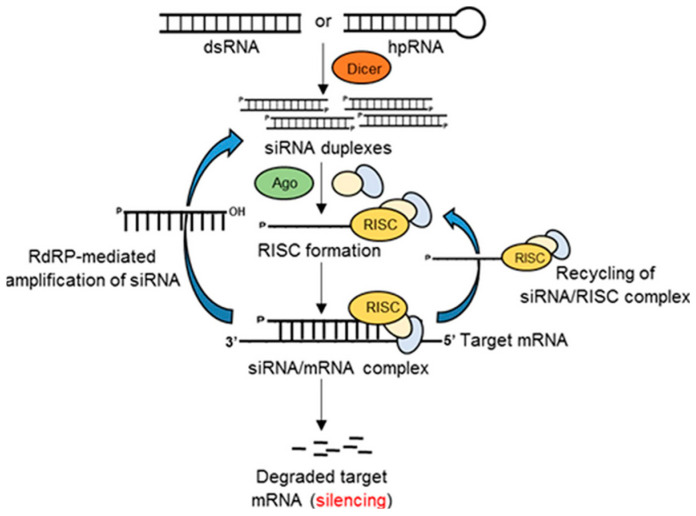
Graphic of the mechanism of the RNAi in eukaryotic cells [63].

Gene silencing, or RNA interference (RNAi), has reformed genetics. With time, it has become clear for researchers that RNAi has a central role in the regulation of diverse processes in animals and plants [56,57]. Genes are be expressed under normal situations, but they can be switched off by a certain apparatus in the cell [58]. RNAi occurs in all eukaryote organisms. It is a mechanism for silencing gene expression; namely, it inhibits the translation of RNA [56]. This new, reliable method transforms experimental biology from single-celled protozoa to mammals. It has several advantages over other nucleic-acid-based methodologies and, as a result, it is recently the most broadly applied gene silencing technique in functional genomics [59]. The mechanism requires an endonuclease enzyme called dicer. Dicer is a cytoplasmic RNAse III enzyme with endonuclease activity that cuts the long double-stranded RNA (dsRNA) or hairpin RNA (hpRNA) into short fragments of 20–25 base pair nucleotides. These generated short fragments are called small interfering RNA (siRNA), which are duplexes after the cleavage, but then are unwound into two single strands. One of the two strands is degraded in the cytoplasm by subsequent cellular proceedings. This strand is called the passenger strand. The other strand is the guide strand, and it incorporates with Argonaute (Ago) and other proteins to form an RNA-induced silencing complex (RISC) [60]. The other three enzymes in this multiprotein complex are helicase, nuclease-ribonuclease and RNA-dependent RNA polymerase (RdRp). Each enzyme has a specific function: helicase unwinds the double-stranded siRNA, whereas nuclease-ribonuclease cuts mRNA and RdRp extends the silencing signal. Ago protein, the catalytic component of the RISC, cleaves the target mRNA strand. The guide siRNA of the siRNA/RISC complex leads the gene silencing to target mRNA; thus, results the degradation of the target transcript or inhibition of translation (Figure 6). Consequently, the protein synthesis is interrupted. The elements of the siRNA/mRNA complex can be reused. RISC or siRNA duplexes will be generated and amplified by the act of RdRp [56,61,62]. 

Gene silencing has two types, which regulate the endogenous genes at the transcriptional level and the post-transcriptional level [64]. In transcriptional gene silencing, histones are modified, generating an environment of heterochromatin around a gene. Thus, the process of transcription is not possible because the gene is inaccessible to the transcriptional procedure after the modification [64,65]. In the post-transcriptional gene silencing, the mRNA will be inhibited accordingly, preventing translation. Furthermore, it will initiate the degradation of mRNA [56,66]. The RNAi is a natural mechanism of post-transcriptional gene silencing [66]. The RNAi mechanism has two main types with small differences. They are mediated by either siRNA (with 21–23 nucleotides) or dsRNA that is longer and may generate a great number of siRNA [67,68]. The dsRNA produces a more varied pool of efficient siRNA combined into RISC complexes than the shorter siRNA [69]. The process of gene silencing protects the genome from invading viruses and transposons. It is probably a part of an ancient immune system protecting the genetic material from infectious gene elements [70]. In addition, it executes cellular functions to survival, health and development and it can supply an innovation for gene therapy [57,71]. 

#### 1.2.5. Applications (Targeted Gene Modification in Aquatic Animals)

Regrettably, there are numerous infectious pathogens that have a negative effect on the fish food industry. These infectious pathogens should be detected and characterized, and treatment strategies with modern and up-to-date techniques should be developed against them to outpace great disease outbreaks [72]. Furthermore, the effectiveness, production, efficiency and wellbeing of cultured fish could be improved with enhanced disease resistance transgenic fish.

Fish are a potential model with several advantages as bioreactors in comparison to mammals. They have a short generation interval and are easy and low-cost to maintain, even with enormous numbers of individuals and high-density culture. In addition, mammalian viruses and prions are not found in fish populations. Some examples are now available representing the potential of fish as bioreactors for medical products. Additionally, various developed complexes can be applied in fish spawning [45]. 

In the following section, we will summarize the above-mentioned different applications of gene modifications in fish medicine.

## 2. Gene Editing in Fish Farm Species Using CRISPR/Cas9 and other Gene Editing Tools

Over 70 aquatic fishes’ genomes have been deciphered during the last few decades. Some end-products in aquaculture created by CRISP/Cas will one day be appraised for commercialization. Some notable advances being developed in several fish species include sterility, disease resistance, pigmentation and improved growth. Gene editing methods have the ability to provide far-reaching keys to challenges in aquaculture [73]. 

### 2.1. Gene Editing in Fishery Science

Zebrafish is widely used as a model organism to study and investigate genetic modifications. It is an excellent model of vertebrate diseases and development because of its fast growth, transparent embryos and its comparatively facile forward genetics. Researchers have used gene editing tools in zebrafish to obtain answers for important problems in fish genetics, reproduction, toxicology, drug-receptor and host-pathogen interaction with favorable results. CRISPR/Cas9 has been successfully used in the development of gene modification in diverse fish species, like Atlantic salmon, medaka, zebrafish and tilapia [74,75,76,77,78,79]. A study presented an effective targeted and heritable gene editing method using CRISPR/Cas9 in Nile tilapia (*Oreochromis niloticus*). The mutation in two genes (*foxl2* and *dmrt1*), induced by CRISPR/Cas9, were successfully transmitted through the germline to the *F*1 generation [80]. Moreover, this study shows the usefulness of the CRISPR/Cas9 technique with high efficiency in non-model species, like genetically engineered tilapia and other aquaculture fish.

### 2.2. Gene Editing in Mono-Sex Population

Gene editing tools propose various nature-friendly ways to produce mono-sex populations. Sexual dimorphism is a traditional feature. There is also a systematic difference in plenty of fish species, which is presented in body growth. As an example, male tilapia grow faster than females, while female rainbow trout (*Oncorhynchus mykiss*) and Indian major carps grow faster than their male partners. The difference in growth rates can be evaded with the production of a mono-sex population, which could raise the yield rates per unit of area. Additionally, it can reduce the threat of unwanted reproduction of prolific fishes in the wild through the production of a mono-sex population. With targeted nucleases, it became possible to produce mono-sex and sex-reserved fishes by a direct route disrupting the sex-determining genes without provoking any significant influence on biodiversity. Knockout of the genes in tilapia that determine the sex of the female (with the XX sex-determining chromosome), such as *fox12*, *sf-1* or *cyp19a1a*, were attained via targeting testicular development. Another way to process sex reversal was achieved via organization of androgen or gynogen hormones; however, this method leads to massive problems, like bioaccumulation, biomagnification and other problems with water quality and biodiversity [9,52]. 

Medaka fish (*Oryzias latipes*) is particularly useful in studying reproduction because of the availability of its genetic information on the regulation of its reproduction [81,82,83]. In a study, the TALEN technique was used to generate the target gene knockout (KO) for gene *gnrh1* (hypophysiotropic GnRH) [84]. *lhb* and *fshb* (vital subunits for the LH and FSH hormones, separately) was used in medaka (*Oryzias latipes*) [85]. The study reported that TALENs successfully cut the targeted sites of the corresponding genes. TALEN-induced disruption of the gnrh1 causes female infertility due to anovulation. All the male KO medaka were fertile, and their testes normally reached maturity. The infertility of the gnrh1 KO female medaka clearly verified that GNRH1 has an important role in the regulation of reproduction in females.

### 2.3. Gene Editing in Sterility of Fish

**Figure 7:**
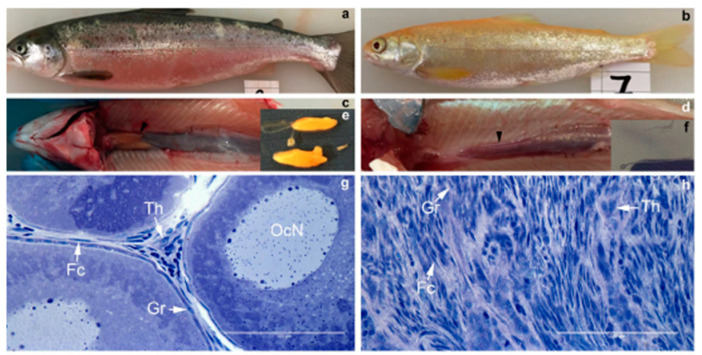
Morphology and histology of one-year-old dnd/alb KO and a control Atlantic salmon. Figure 7 presents the control fish on the left side (**a**,**c**,**e**,**g**). The *dnd*/*alb* KO fish is presented on the right side (**b**,**d**,**f**,**h**). Fish b is a female *dnd*/*alb* KO fish. D and f show the gross morphology of the female *dnd*/*alb* KO in comparison to the gross morphology of the control female (**a**,**c**,**e**): the lack of the ovarian bulb in comparison to the control (**e**); g and h show the histology of the female gonad in *dnd*/*alb* KO fish (**h**) in comparison to the control ovary (**g**). Abbreviations: Th—theca cell; OcN—oocyte nucleus; Gr—granulosa cell; Fc—fibrocyte [89].

Sterility in fish could easily be tackled with targeted nucleases controlling unwanted fish reproduction in predatory and weed species, as well limiting the establishment of exotic and transgenic fishes in the wild if they accidentally escape from separated milieu-like ponds and flow-through systems. The production of sterile fishes can help us overcome such problems. For example, sterile channel catfish (*Ictalurus punctatus*) are produced by using ZFN technology, and by disrupting the subunit gene of the pituitary luteinizing hormone. The sterile catfish reduced the potential environmental and ecologic hazards; therefore, the catfish industry could profit. This was the first sterilisation using a ZFN mechanism in aquaculture, as well as the first effective gene editing of channel catfish [9,86]. A major problem of fish farming are the escaped Atlantic salmon (*Salmo salar* L.), as they are cultured in open sea cages during the growth period [87]. Sterile fish, namely germ cell-free salmon, could reduce this problem by stopping the introgression, the gene flow between domesticated salmon into wild stocks [88]. A germ cell-free salmon was produced in F0 by using CRISPR-Cas9 to knockout the dead end (*dnd*) gene [89]. Dnd allows the survival of germ cells. The knockout of the *dnd* gene in mammals leads to an all-male offspring [90]. CRISPR/Cas9 was used to influence the pigmentation in salmon(Figure 7), namely the targeted knockout of the slc45a2 (*alb*) pigmentation gene, which leads to a completely albino phenotype [78]. 

The *dnd/alb* KO mutant Atlantic salmon were produced through double allelic mutations using CRISPR/Cas9. As a result, the fish were completely lacking pigmentation and were devoid of germ cells in F0. The study showed that the biallelic KO sustains with high probability in long-life-cycle-species, which prohibits the generation of F2 [89]. In addition, the germ cells are not required for female sex differentiation, but may be required for establishing a normal structure in the ovaries in Atlantic salmon. This study demonstrated for the first time that CRISPR/Cas9-mediated KO of *dnd* leads to a complete loss of germ cells in F0 generation in fish species [89].

### 2.4. Gene Editing in Reproduction

The *Kiss1/Gpr54* system (kisspeptin-encoding gene—*Kiss1* [91] and its G protein-coupled receptor 54—*GPR54* [92]) has a central role in the regulation of reproduction in most vertebrates [93,94,95]. These systems have also been identified as multiple *kiss1*/*gpr54* paralogous genes (*kiss/kissr*) in non-mammalian vertebrates, which is different from mammals. During a study [96], zebrafish *kiss1*-/-, *kiss2*-/- and *kiss1*-/-; *kiss2*-/- mutant lines together with *kissr1*-/-, *kissr2*-/- and *kissr1*-/-; *kissr2*-/- mutant lines were generated using an optimized TALEN restriction enzyme. The result clearly showed that the spermatogenesis, folliculogenesis and a reproductive potential are not damaged in all of these mutant lines. The fish were normal and fertile in both sexes. Furthermore, the data indicated that *kiss/kissr* systems are not required for zebrafish reproduction, signifying that the *kiss/kissr* systems represent unnecessary roles for reproduction in definite non-mammalian vertebrates. It is also shown that mammals and fish have developed different strategies for neuroendocrine control of reproduction.

### 2.5. Gene Editing in Fast-Growing Fishes

Several endemic cold water fish species have a slow growth rate because of their genetic nature and physiology and environmental limitations of their surroundings. Cold water fishes have an excellent virtue: they can live in stagnant water (ponds), while other species demand continuous clean and well-aerated water. With the help of targeted nucleases, the expression of growth-promoting genes could be increased. Furthermore, the gene inhibiting the skeletal muscle growth could be knocked out [9]. In one study [97], the gene coding from myostatin (suppressor of muscle growth) in common carp (*Cyprinus carpio*) was disrupted by CRISPR/Cas9. As a result, the mutated fishes grew considerably more muscle cells, and showed larger phenotypes in the F0 generation, therefore the carp genes were successfully targeted. Analogous methods have been used to increase the production of slow-growing cold water fishes, like snow trout [9]. 

### 2.6. Gene Editing in Ornamental Fishes

The production of ornamental fishes with desired colors and pigmentation can also be realized by targeted genome editing tools. Thanks to ZFN, TALEN and CRISPR/Cas9 techniques, the mutation of golden genes resulted in the making of light-colored eyes that are inheritable up to the F1 generation [76,98,99]. Somatic and germline disruptions of genes in zebrafish (*Danio rerio*) were accomplished with the use of zinc finger nucleases (ZFNs). The designed ZFNs targeted the golden and no tail/*Brachyury (ntl)* genes of the zebrafish. Thanks to the injection of ZFN-encoding mRNA into the one-cell embryos, a significant percentage of the animals had different mutations at the ZFN-specified locus in the fish and presented with the corresponding awaited loss-of-function phenotypes. The results of this study confirm that ZFN technology is applicable to produce heritable mutant alleles precisely and professionally at the loci of interest. This study also suggests that this method may be essential in several organisms that allow mRNA delivery into the fertilized eggs [76]. 

**Figure 8:**
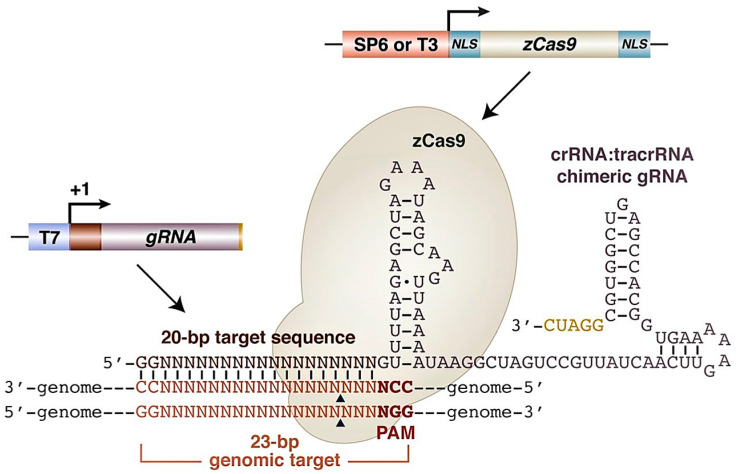
Genome editing with CRISPR/Cas9 nuclease system in zebrafish (*Danio rerio*) [99].

The CRISPR/Cas nuclease system also represents a highly effective gene knockout method in zebrafish. With custom guide RNAs and a zebrafish codon-optimized Cas9 enzyme, the researchers efficiently targeted the correspondent transgene *Tg*(*-5.1mnx1:egfp*) (Figure 8) [99]. Furthermore, four endogenous loci were also successfully targeted (*tyr*, *golden*, *mitfa* and *ddx19*). The high rate of the mutagenesis proves that most cells contained biallelic mutations. In four of the five target cases, recessive null-like phenotypes were observed, denoting the high level of the biallelic gene disruption. Additionally, effective germ-line transmission of the Cas9-induced mutation was noticed. The created nuclease system was injected into one-cell-stage embryos to induce RNA-guided targeted DNA DSB through the Cas9 enzyme. The result of this research also indicates that five genomic locations can be targeted together at the same time, with outcomes in multiple loss-of-function phenotypes in the same vaccinated zebrafish.

Figure 8 shows the mechanism of the CRISPR/Cas9 system in the above-mentioned study [99]. The nuclease system consists of a dual NLS-tagged zebrafish codon-optimized Cas9 protein with a single crRNA:tracrRNA chimeric gRNA. The mix was injected into one-cell-stage embryos to induce RNA-guided targeted DNA DSB through the Cas9 enzyme. Both components together comprised a 20-bp target sequence (dark red) to a PAM site of NGG and were first produced as RNAs by in vitro transcription from the SP6 or T3 (for Cas9) or T7 (for gRNA) promoter [99]. 

### 2.7. Gene Editing in Pigmentation

**Figure 9:**
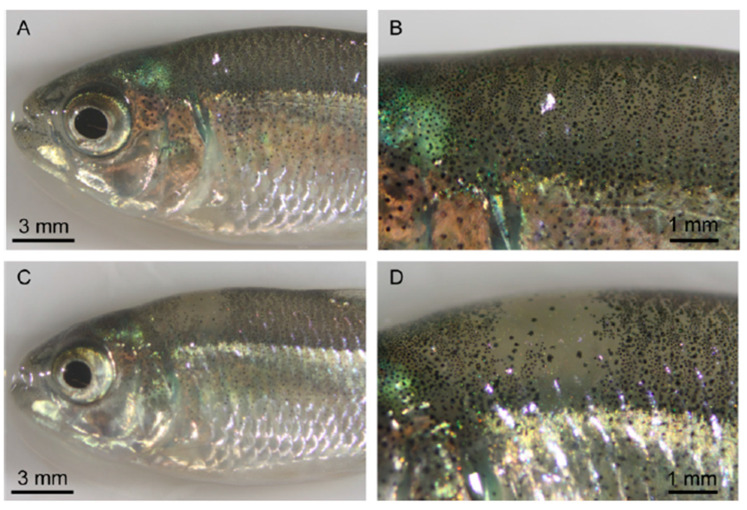
Interpretation of pigmentation in surface fish in oca2-injected F0s. The panels in Figure 9 present the following analysis. Panel (**A**) shows the pigmentation in an uninjected fish, whereas panel (**B**) depicts a close up of the dorsal region of uninjected surface fish from picture (**A**). Panel (**C**) illustrates the pigmentation in a 400 pg *oca2* exon 9 injected F0 surface fish, whereas panel (**D**) shows a close up of a pigmentation patch lacking the melanin pigmentation from picture (**C**) [100].

Another study represented a successful gene editing process with the help of TALEN in teleost fish, namely in the cavefish (*Astyanax mexicanus*) [100]. This fish species is a brilliant model for studying the genetic basis of evolution. The study used designed TALEN to target two genes in the cavefish (*Oculocutaneous albinism 2* (*oca2*) and melanocortin 1 receptor (*mc1r*)) that contain coding sequences responsible for reduced pigmentation (Figure 9). The results show that the genes of cavefish can be mutated using this technique and that the modification is noticeable in the fish. Specifically, the induced mutations in oca2 result in the mosaic-patterned loss of melanin pigmentation, namely the lack of melanin-producing melanophores in the regions that were lighter in appearance under the microscope. They appear as albino patches in F0 founder fish, signifying biallelic gene mutations in F0s, permitting us to evaluate the role of this gene in pigmentation. Apparent differences in the phenotype were not observed in the pigmentation of *mclr*-TALEN-injected fish compared to non-injected familial fish. This process demonstrates that TALEN has the potential to create mutations at specific locations in Astyanax mexicanus. This organism has become a dominant model system for researching the genetic basis of evolution in an extreme location, the cave. This study also shows that TALEN has an advantage over CRISPRs for this type of experiment. TALEN can be used at potentially any site in the genome. However, only limited sites can be targeted through CRISPRs, as they require a PAM sequence [101]. 

**Figure 10:**
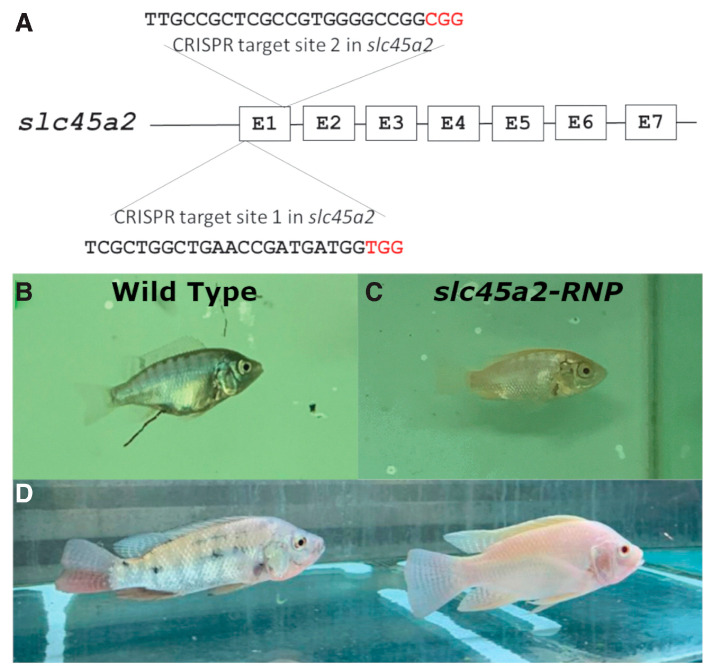
Different phenotypes between wild adult and slc45a2 mutant adult Nile tilapia Figure 10 shows the phenotypic analysis of tilapias after microinjected at the single-cell stage with RNPs. (**A**) shows Nile tilapia zygotes were containing *slc45a2*-*exon1*-specific gRNAs 2 and 3. (**B**) shows the embryo with a normal gray-black pattern and dark eyes at 1-month post fertilization. (**C**) shows *slc45a2*-RNPs-injected mutant fish with 97–99% loss of melanin in the skin and no melanin in the eye. (**D**) shows post sexual maturation, F0 mutant displaying a complete loss of melanin [102].

A recent study describes the generation of stable and heritable red tilapia phenotype through induced loss-of-function mutations in the *slc45a2* gene of Nile tilapia (*Oreochromis niloticus*) [102]. The solute carrier family 45 member 2 (*slc45a2*) is a membrane-associated transporter protein that mediates melanin biosynthesis and is evolutionarily conserved from fish to humans. To achieve this purpose, the *slc45a2* gene in the fish was identified and highly specific gRNAs (*gRNA2* and *gRNA3*) were designed against this gene. Tilapia zygotes at the single-stage cell received multiple microinjections of slc45a2-specific ribonucleoproteins (RNPs). As a result, the microinjection induced up to 97–99% albinism, which generated a solid-red phenotype, including loss of melanin in the eye. Mutant alleles were carried in all the injected fish with variable mutagenesis efficiencies, presented by the next-generation sequencing of the injected zygotes. The sequencing analysis of gDNA from the F0 albino mutant and its heterozygous F1 offspring demonstrated that the new *slc45a2* mutant alleles with a red phenotype in Nile tilapia are stable, trackable and heritable (Figure 10). The study shows that the CRISP/Cas9 technique has applicative potential in *O. niloticus* culture.

### 2.8. Gene Editing in Growth

Most research work has concentrated on the transfer of GH (growth hormone) genes. Enhancement of growth (size and rate) has jumped from 0% to an incredible 300% under certain circumstances [45]. The myostatin (MSTN) gene is a regulator of skeletal muscle growth in all vertebrates and controls myoblast differentiation in vitro [103]. Modifying the myostatin via gene knockout or overexpression of inhibitors increases muscle mass, in particular [104,105]. In the study by Khalil et al. [106], a successful targeting of the muscle suppressor gene MSTN in channel fish (*Ictalurus punctatus*) through the CRISPR/Cas9 system was presented. A CRISPR zygote microinjection was used to knockout the MSTN gene and determine the effects of knockout on growth. In the target protein-encoding site of MSTN, high rates of mutagenesis were induced. Mutated fry had more muscle cells than the control group, and their average body weight increased by 29.7% 40 days after the microinjection. A large percentage of the embryos were mutated within the target sites, and no mutations were detected nearby or outside the target site.

The results of this study exhibit that, with the CRISR/Cas9 tool, channel fish genomes can be edited very efficiently, and that, with this technique it will be possible to ease the genetic improvement and functional genomics of channel catfish [106]. Thanks to this approach, it is possible that growth-enhanced channel catfish will be produced, which will increase the productivity.

### 2.9. Gene Editing in Body Configuration

The transgenic modification of the nutritional characteristics of fish is already probable via transgenesis, which could be advantageous for customers [45]. Zebrafish transfected with B-actin-salmon desaturase genes have increased levels of omega-3 fatty acids, eicosapentaenoic acid (EPA) and docosahexaenoic acid (DHA) in their meat. Another analogous result was verified with the same transgene transfer to common carp and channel catfish. The expression of these transgenes was certified [45]. 

### 2.10. Gene Editing in Oomycetes

The oomycete *Aphanomyces invadans* causes epizootic ulcerative syndrome (EUS) in many fish species. It leads to mass mortality in cultured and wild fish worldwide and generates a huge economic impact [107,108]. Extracellular proteases produced by this oomycete initiate the EUS disease process [109]. One study [110] identified the secreted proteases from *A. Invadans* utilizing SDS-PAGE and mass spectrometry, followed by BLASTp analysis. Three prominent protein bands were shown through SDS-PAGE and identified via spectrometer. The proteolytic activity of these proteases was assessed on casein and fish immunoglobulin M (IgM) of rainbow trout and giant gourami (*Osphronemus goramy*). The secreted proteases were able to degrade the casein and IgM in both of the fish species. The activity of the antiprotease of the fish serum was also explored. The findings presented an inhibition of secreted proteases using several protease inhibitors to reduce the proteolytic activity. Furthermore, the results suggest that the extracellular proteases could potentially affect *A. invadans* as a virulence factor. This study offers further functional investigations on the role of the identified proteases in EUS pathogenesis and paved the way for using genome editing tools, such as CRISPR/Cas9 nuclease, for development of the drug against this disease [110,111].

The study utilized three single guide-RNAs (gRNA) to target the oomycete *A. invadans* serine protease gene [111]. This oomycete is a group of parasites and the primary causal factor for epizootic ulcerative syndrome (EUS). The zoospores develop in the sporangium in fish tissues and create dermal lesions presenting as deeper ulcers, red spots or blackish burn-like marks [112,113,114,115]. Secreted protease, especially genes from serine protease secreted by *A. invadans*, which have already been identified and used as the targeted gene for gene editing [110,111]. The CRISPR/Cas9 system was used to target these genes in a test to investigate its function in EUS. Three dwarf gourami (*Trichogaster lalius*) groups were intramuscularly injected with three different suspensions to examine the effect of the edited genes on the virulence of the oomycete. One group received non-treated *A. invadans* zoospores, another group received RNP-treated *A. invadans* zoospores and the third group was inoculated with autoclaved pond water as a negative control. During the 30 days of the in vivo experiment, the group with the RNP- treated zoospores and the control group did not present any clinical signs; the PCR did not extend into the DNA of *A. invadans*. A histological analysis also did not find any infiltration or necrosis of the muscle tissues in these two groups. On the contrary, the positive control group showed characteristic symptoms, including ulcers in the skin and muscles and swelling and presented *A. invadans* hyphae.

Summarily, this study established successful gene editing via CRISP/Cas9, which prevented the production of serine protease. With this promising tool, it is practicable to study oomycetes and secreted proteases in order to control EUS; it may also help in the development of drugs against this pathogen.

## 3. Gene Silencing in Fish Medicine

The RNAi tool has been commonly utilized to understand and examine gene function in aquatic diseases [116,117]. It is also appropriate for the development of therapies for viral diseases in livestock and aquatic creatures. In addition, it represents one of the newest and most promising methods in antiviral medicines and therapeutics [118]. Almost all the studies applying RNAi tools in fish have been successfully performed in zebrafish (*Danio rerio*), which is a valuable fish model organism for aquaculture and biomedicine applications [59,119] (Table 2).

### 3.1. Gene Silencing in Viral Disease of Fish Medicine

With RNAi-based therapies for viral diseases, invertebrate, vertebrate and human pathogens can also be treated [120]. The inhibition of gene transcription and the study of viral replication were completed by Gotesman et al. [121]. SiRNA molecules targeted the nucleoprotein “N” and phosphoprotein “P” transcripts to inhibit in vitro replication of the spring viraemia of the carp virus, (SVCV), and they were tested in a cell line from *epithelioma papulosum cyprini* (EPC). This virus belongs to the *Rhabdoviridae* family of viruses and causes severe loss in carp farms. The study showed that using siRNA to inhibit the of SVCV-N and SVCV-P genes’ expression reduced SVCV replication. In another study, the in vitro viral replication of cyprinid herpesvirus-3 (CyHV-3) was inhibited by (si)RNA in common carp brain cells (CCB cells). This virus causes high mortality rates both in common and koi carp (*Cyprinus carpio* L.). The siRNAs were meant to target either thymidine kinase (TK) or DNA polymerase (DP) genes, which are the codes of transcripts in DNA replication. The treatment with siRNA shows that TK or DP genes reduced the release of viral elements from contaminated CCB cells; that is, siRNA inhibited the viral replication [122]. CyHV-3 is most successfully inhibited via the RNAi-mediated gene silencing technique when multiple viral genes are targeted [122,123]. 

### 3.2. Gene Silencing in Parasitic Disease of Fish Medicine

The treatment of parasitic diseases with the RNAi mechanism has shown promising results as well. The study by Saleh and co-workers demonstrated that siRNA could be used to knock down the expression of specific genes of *Heterosporis saurida*, a parasite of the lizardfish (*Saurida undosquamis*) [124]. SiRNAs were designed to inhibit the ATP/ADP antiporter 1 and methionine aminopeptidase II genes and tested in an in vitro cultivation model. This study concluded that siRNA reduced the targeted gene transcription and spore counts of *H. saurida*, concluding that this process is an advanced development for inhibiting this microsporidian parasite. Salmon whirling diseases are caused by the cnidarian myxozoan parasite *Myxobolus cerebralis*, whose one alternative host is an invertebrate oligochaete, *Tubifex tubifex* [125,126]. In the study from Sarker and El-Matbouli [127], the researchers used targeted siRNA-mediated gene silencing for MyxSP-1 serine protease in vivo in *M. cerebralis*-infected oligochaetes, providing an intervention strategy in salmonid whirling disease. During the research, *T. tubifex* was soaked in a special solution with fluorescently labelled siRNA and, as a result, it was observed that siRNA was taken up from *T. tubifex*. The fluorescence was detected in the body of the oligochaetes. In addition, the researchers observed knockdown in MyxSP-1 mRNA expression.

Another study demonstrated that *T. tubifex* soaked in a solution holding dsRNA targeting the *MyxSP-1* of the *M. cerebralis* inhibited the myxozoan parasite from infecting the rainbow trout fry host [128]. The specific-pathogen-free rainbow trout fry were immersed in water inhabited by live siRNA-treated *T. tubifex*. The siRNA treatment with *MyxSP-1* presented maximum significant knockdown, and the salmonids did not show signs of salmonid whirling disease. These results show the proof of successful RNA-based therapy in vivo against this parasitic infection in salmons.

### 3.3. Gene Silencing for Gene Function Studies in Fish Medicine

The successful inhibition of zebrafish gene expression via a short hairpin RNA (shRNA)-mediated process was presented in another study [129]. ShRNAs originate from longer double-stranded (ds) precursors, and they can be used for gene silencing because they can post-transcriptionally prevent the expression of complementary RNA [130]. Two genes (*wnt5b* and *zDisc1*) were used for the test, each with a similar phenotype in both genetic mutants and morphants. The results show that shRNAs inhibited *wnt5b* expression and targeted *zDisc1* effectively and specifically. In summary, shRNAs decrease endogenous RNA levels in zebrafish gene expression. Wang and co-workers studied the knockdown of the green fluorescent protein (*gfp*) and no tail (*ntl*) gene expression by in vivo-transcribed short-hairpin RNA (shRNA) with a T7 plasmid system in zebrafish (*Danio rerio*) embryos [52]. The T7RP expression vector and the T7shRNA vectors target these two genes, respectively. The study was based on the specific identification of the T7RP to T7 promoter, and the transgenic zebrafish line stably expressing T7RP was recognized. Additionally, the shRNA vectors that targeted the foreign *gfp* gene and the endogenous *ntl* gene were created.

**Figure 11:**
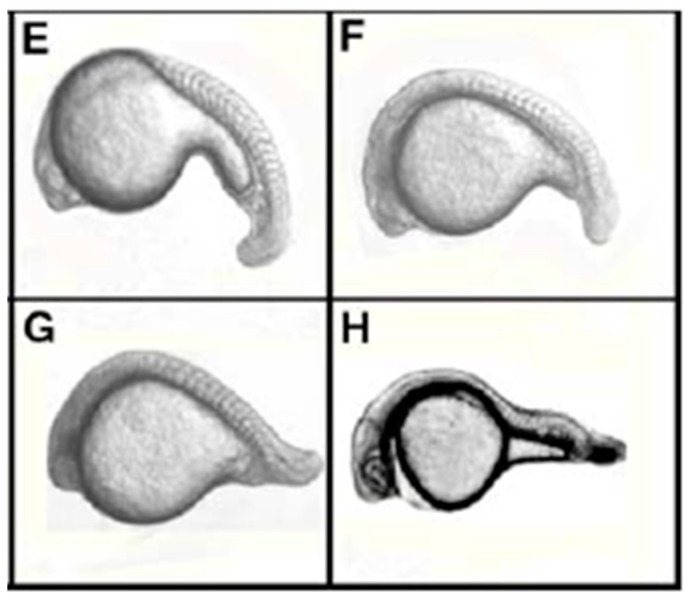
(**E**,**H**) shows the phenotype that appeared at the 25-somite stage of zebrafish embryos. The similar ntl phenotype was also detected in 14% (11/77) of the embryos (**F**,**G**). E illustrates a wild-type zebrafish embryo and H presents the ntl mutant [71].

Ultimately, the shRNA constructs (pT7Bmp2b) were injected into the F3 embryos of the pCMVT7R transgenic line. The results reveal that the T7 transcription system could function to drive the expression of shRNA in zebrafish embryos and eventuate the gene knockdown effect (Figure 11).

**Figure 12:**
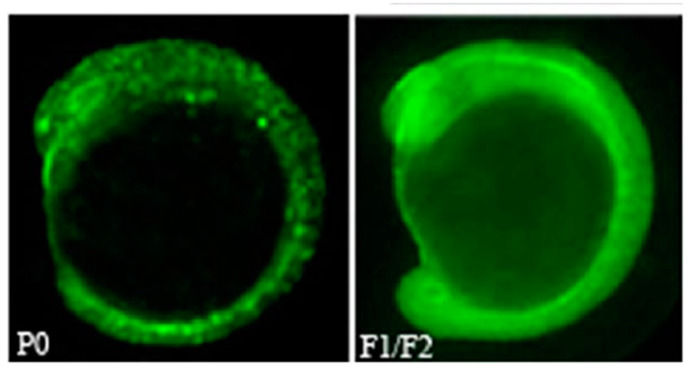
Gfp gene expression in a pCMVT7R transgenic zebrafish embryo of mid-somite stage. The P0 embryo expressed the gfp gene mosaically; F1/F2 one expressed the gfp gene uniformly in the whole embryo [71].

Another study [131] used two different siRNA techniques to demonstrate a highly efficient gene knockdown method in three different zebrafish lines, ZFL, SJD and ZF4 cell lines, which was derived from adult and embryonic zebrafish (*Danio rerio*). Different zebrafish genes, *lamin A*, *lamin B2*, kinesin-related motor protein *Eg5* and exogenous GFP (*eGFP*) were chosen as the target genes to be silenced. Knockdown of the target genes with specific phenotypes was noted from previous studies for homologous siRNA in mammalian cells. In contrast, injection of *lamin A*, *GL2* (control) and *eGFP* siRNAs into zebrafish embryos influenced the morphology and led to morphological defects, abnormal development and the early death of most of the embryos (Figure 12).

This study presented, for the first time, that the cellular RNA interference mechanism works in *Danio rerio* cell lines. Moreover, it demonstrated that the active RNAi machinery of a specific gene in cell lines is possible.

### 3.4. Gene Silencing in Oomycetes

**Figure 13:**
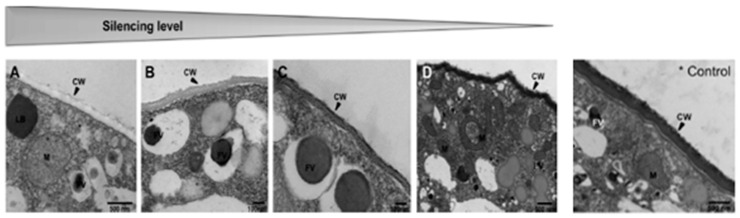
Effect of silencing of SpTyr gene on the cell wall of Saprolegnia parasitica [133]. Figure 13 describes the gene expression level of SpTyr-silenced lines using TEM. This method exposes an electron-dense layer in the cell wall (CW) of the sporangia of the control lines (*) and a non-silenced line (**D**). The pictures (**A**–**C**) show the decreased electron-dense layer in the cell wall of the sporangium with decreased levels of SpTyr-expression [132].

The first application of gene silencing in a relevant aquaculture pathogenic oomycete, *Saprolegnia parasitica*, was described in the study by Saraiva et al. [132]. The gene of tyrosinase, *SpTyr*, is necessary for the melanin biosynthesis of this fish pathogen. It is involved in pigment formation and a decrease in the expression of this gene can cause detectable changes in the phenotype. Different *S. parasitica* lines were treated with SpTyr-dsRNA. After tyrosinase gene silencing, the melanin production was reduced and the tyrosinase activity decreased between 38% and 60%. The SpTyr-silenced lines exhibited less pigmentation in developing sporangia, which was sporadically modified; abnormal morphology; and a less electron-dense cell wall (Figure 13). This work demonstrated that gene silencing via RNAi is a suitable method to functionally identify genes in *S. parasitica*.

### 3.5. Gene Silencing in Crustaceans

The limited information regarding the gene content of crustaceans and the absence of tools for genetic manipulation has made it challenging to follow the mechanistic basis for dsRNA in crustaceans. Expanding our knowledge about genomics and proteomics in crustaceans should supply the key to solving the molecular mechanism in this new occurrence. Presently, few studies have explained the RNAi method and recognized its practical use in the study of gene function in crustaceans [59]. Another study declares that the RNAi method is widely utilized as a technique to examine gene function and develop antiviral agents to fight viral infections in invertebrate animals [133]. 

The first metazoan in which the gene silencing process was registered was a nematode *Caenorhabditis elegans* [134]. The nature of RNAi presented in this animal the capability of cells to notice and interiorize extracellular dsRNA to initiate intracellular procedures of gene silencing in vivo [135,136]. Gene silencing can be generated in different ways; for instance, via feeding, injection or transgenic expression of dsRNA molecules [137]. Table 3 shows a summary of the RNAi method in crustaceans from one study [59]. 

#### 3.5.1. Gene Silencing in Viral Diseases of Crustaceans

Recently, three unrelated viral diseases in shrimps have been target inhibited with the dsRNA technique: white spot syndrome virus (WSSV), yellow head virus (YHV) and Taura syndrome virus (TSV). This section describes the successful studies that address them. In a study by Tirasophon and co-workers, primary cultures of black tiger shrimp (*Penaeus monodon*) lymphoid ‘Oka’ cells were used to verify the inhibition in the viral replication of YHV through RNAi-mediated gene silencing [67]. In vitro transcribed dsRNA of YHV helicase (*hel*), protease (*pro*), polymerase (*pol*) and structural viral genes *gp116* and *gp64* were transfected into a shrimp Oka cells culture, and the morphological change was investigated under a microscope. As a result, it was found to inhibit YHV replication. DsRNA was more effectively targeted to the non-structural genes (protease, polymerase and helicase) of YHV than the structural genes in suppressing the viral replication. The targeted structural genes (*gp64* and *gp116*) had the least inhibitory effect on viral replication. This study demonstrated that the dsRNA controlled the primary cell culture of *Penaeus monodon* to protect against YHV infection. In addition, it shows the first proof that RNAi-mediated gene silencing also works in shrimp cells.

The YHV shrimp virus causes significant economic damage and production losses in farmed penaeid shrimp [138,139]. Another study used dsRNA-mediated RNA interference silencing to specifically downregulate the pmYRP65 message [140]. The 65-kDa receptor protein by YHV therefore inhibited the whole virus entry in the *Penaeus monodon* cells. A primary cell culture from the lymphoid (Oka) organ of *P. monodon* was then applied to target this viral infection. This report marks the first identification of an invertebrate *Nidovirus* receptor, namely *pmYRP65*. The antibodies against this protein and the down regulation of the *pmYRP65* message via RNAi are able to inhibit the entry of yellow head virus into Oka cells, recommending that the protein identified is certainly a YHV receptor protein, the 65-kDa protein. In the absence of the message, the lymphoid organ cells were shown to be refractory to infection with this virus, proving that *pmYRP65* acts equally as a receptor protein for YHV.

**Figure 14:**
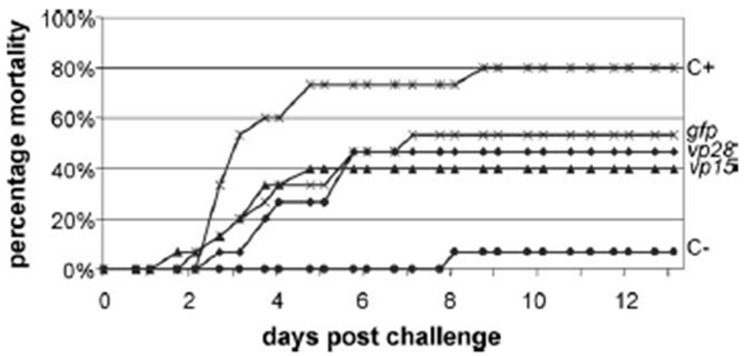
Time–mortality graph of shrimps (Penaeus monodon) injected with siRNAs [141].

White spot syndrome virus (WSSV) gives rise to mortality and causes serious losses in commercial shrimp farms worldwide because of the current intensity of aquaculture practices. An alternative and effective methodology to prevent this infection in shrimp could be the utilization of an RNA interference. Shorter 21-nucleotide siRNAs with homology were investigated in the WSSV and either *vp15*, *vp28* or *gfp* genes give a sequence-specific interference and response in the shrimp *Penaeus monodon* in one study [141]. *Vp15* is a basic DNA-binding protein of WSSV [142]; *vp28* is a main WSSV cover protein that probably also participates in systemic virus infection [143]; *gfp* siRNAs are useful for nonspecific control of siRNA effects. The intramuscular injection of the *vp28* and *vp15* siRNAs induced an important reduction in shrimp mortality upon WSSV infection, but no such specific difference was observed in the reduction when the control *gfp* siRNA was used (Figure 14). Both shrimp injected with siRNAs and large dsRNA molecules showed a sequence-independent antiviral immunity.

Figure 14 presents the above-mentioned study [142]. Each shrimp was vaccinated with 10 µM siRNAs or buffer (C+ and C−). Twenty-four hours after the injection, they were challenged with WSSV or injected with buffer (C−). Their collective mortality rate is shown against a day after the challenge (*n* = 15).

Another study used freshwater crayfish (*Pacifastacus leniusculus*) to experimentally infect with the white spot syndrome virus (WSSV) [144]. Numerous differentially expressed genes were recognized and characterized in this study. The protein, namely anti-lipopolysaccharide factor (ALF), was selected because its transcript levels increased upon viral infection. Quantitative PCR revealed that, in the cell culture of hematopoietic tissue from freshwater crayfish, knockdown of ALF via RNAi caused about 10-fold higher WSSV levels than those treated with the control dsRNA. Accordingly, RNA interference experiments with ALF in animals and in cell cultures indicated the protection of ALF against WSSV infection in crayfish, as the knockdown of ALF through RNAi leads to higher rates of viral replication. In other words, the function of the ALF protein in viral propagation is important since its removal via RNAi results in an important improvement of viral replication. Consequently, the report showed that ALF disturbs WSSV dissemination by applying RNAi both in vivo and in vitro. It was the first study to describe RNAi in vitro with a crustacean. It was also the first to identify an endogenous factor interfering with WSSV dissemination in crustaceans. ALF probably has a prominent place in the immune protection against viral infections of crayfish.

#### 3.5.2. Gene Silencing in Bacterial Disease of Crustaceans

Another study [145] indicates that phenoloxidase (PO) is a significant element of the protection of the freshwater crayfish *Pacifastacus leniusculus* against infection by the highly pathogenic bacterium, *Aeromonas hydrophilain*. Phenoloxidase is the terminal enzyme in the melanization cascade, and it takes part in the recognition of and immune defense toward microbial infections in invertebrates. Gene silencing using dsRNA-mediated RNA interference transcript depletion of crayfish prophenoloxidase (proPO) caused several effects: increasing bacterial growth, lower phagocytosis, decreased phenoloxidase activity, lower nodule formation and a higher mortality rate were all observed after infection with this bacterium. On the other hand, if the inhibitory domain of the crayfish prophenoloxidase activating cascade, namely the pacifastin gene, is targeted with dsRNAi, the opposite of the above-mentioned processes occurs. Specifically, it results in lower bacterial growth, increased phagocytosis, increased nodule formation, higher phenoloxidase activity and delayed mortality. In conclusion, the data from this study elucidate that PO is necessary in the freshwater crayfish defense against pathogenic bacterial infection by *A. hydrophila*.

#### 3.5.3. Gene Silencing in Decreased Glucose Levels by Crustaceans

The crustacean hyperglycemic hormone (CHH) is essentially responsible for the regulation of hemolymph glucose levels, growth, molting and reproduction [146]. A study examined the ability of dsRNA to inhibit the function of this hormone in Atlantic Ocean shrimps, *Litopenaeus schmitti*, in vivo [147]. CHH gene silencing was implemented through the injection of CHH dsRNA into the abdominal hemolymph sinuses of the shrimps. After 24 h, the undetectable CHH mRNA levels, the suppression of the CHH gene function and an analogous decrease in hemolymph glucose levels in adult shrimps demonstrated that effective gene silencing had occurred. This study demonstrated for the first time that the dsRNA process works in adult shrimps in vivo, and that it can be used to study gene function in crustaceans.

#### 3.5.4. Gene Silencing in Pleiotropic Effect by Crustaceans

**Figure 15:**
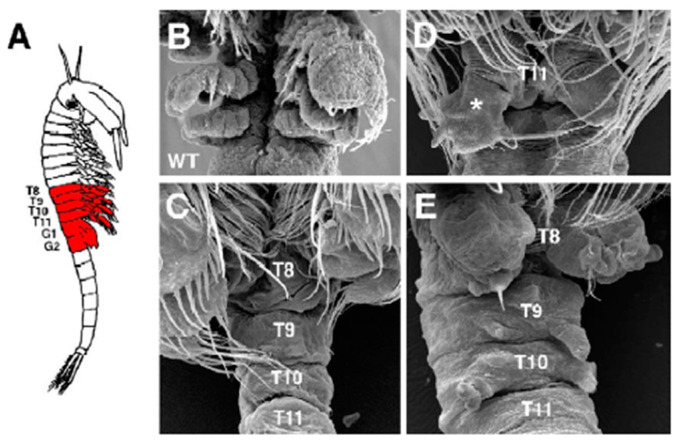
*Artemia franciscana* spalt RNAi modified phenotypes: malformed, rudimentary and missing appendages of different regions of the body. Panel (**A**) shows a highlight of the region of the body where malformed, rudimentary or missing appendages happen, together with posterior thoracic (T8–T11) and genital (G1 and G2) segments. (**B**) shows the scanning electron microscopy (SEM) of the thoracic appendages in a normal individual during mid-larval stages, presenting the characteristic morphology of juvenile, growing phyllopodous appendages. (**C**–**E**) show the SEM of dsRNA- treated individuals in late larval stages: (**C**) an individual with missing appendages in segments T9-T11; (**D**) an individual with a malformed appendage (marked by an asterisk) in the T11 segment, presenting clear abnormalities compared to normal phyllopodous appendages or to juvenile appendages (compare to panel B); (**E**) an individual with rudimentary and malformed (in T9 and T10) or missing (in T11) thoracic appendages. The anterior side is shown in all the panels [148].

A recent study demonstrated that dsRNA caused the knockdown of the expression of spalt genes in the branchiopod crustacean *Artemia franciscana* [148]. The spalt genes have a central effect during development and their function has been nearly joined with the function of *Hox* genes in different contexts. This study examined the role of spalt genes in *Artemia* and found that spalt is expressed in the pre-segmental ‘growth zone’ and in a series of stripes in each of the trunk segments as they appear from the growth zone. The reduced effects of spalt function in *Artemia* were studied via the RNAi method. Due to knocking down of spalt gene expression, pleiotropic effects were created (Figure 15). These effects represented several homeotic transformations in phenotypes, thoracic to genital (T→G), genital to thoracic (G→T) and post-genital to thoracic (PG→T), that are combined with a stochastic depression of *Hox* genes in the analogous segments of RNAi-treated animals. The most common phenotype was characterized by the growth of rudimentary or malformed appendages. In summary, it appears that spalt genes have a possible role in the maintenance of *Hox* gene repression in *Artemia* and in other species. In addition, this result would be advantageous in unravelling the genetic processes that underline a specific evolutionary process in *Artemia franciscana*. 

#### 3.5.5. Gene Silencing in Reproduction of Crustaceans

A report from another study demonstrates that the use of double-stranded RNA elucidates the function of gonad-inhibiting hormones (GIH) in black tiger shrimp (*Penaeus monodon*) [149]. GIH is an essential peptide hormone that regulates reproduction in crustaceans and modulates ovarian maturation by inhibiting the synthesis of vitellogenin (Vg), the precursor of yolk proteins. This study inquired into the cDNA-encoding GIH (Pem-GIH) from shrimp, and its probable role in the eyestalk of *P. monodon* was cloned via RT-PCR and RACE methods. The Pem-GIH transcript was detected in the eyestalk, brain, thoracic and abdominal nerve cords of adult shrimps. With the help of the RNA interference technique, the gonad-inhibiting activity of Pem-GIH was investigated. DsRNA caused a decrease in the Pem-GIH transcript levels both in the eyestalk ganglia and the abdominal nerve cord explant cultures, as well as in female *P. monodon* bloodstock. A functional knockdown study of Pem-GIH through dsRNA was conducted to exhibit the negative influence on Vg mRNA expression in the ovaries of previtellogenic adult females for the first time, providing proof for its role as a gonad-inhibiting hormone in this shrimp species. The study characterized and recognized the Pem-GIH cDNA of *P. monodon* in both biological and molecular viewpoints. As a result, this study proved that dsRNA-mediated gene silencing is a potent tool for the functional study of the genes in crustaceans.

Neurosecretory structures in crustaceans’ eyestalks are produced neuropeptides, namely the crustacean hyperglycemic hormone (CHH), molt-inhibiting hormone (MIH) and gonad-inhibiting hormone (GIH) of the CHH⁄MIH⁄ GIH gene family. These can regulate several processes, such as molting and reproduction [150,151,152]. A study by Tiu und Chan [68] described the production of recombinant protein dsRNA for the eyestalk neuropeptide gene and an RNA interference methodology to study the reproductive function of the molt-inhibiting hormone (MeMIH-B) in female sand shrimp, *Metapenaeus ensis*. Ovary and hepatopancreas explants were cultured in mediums including recombinant MeMIH-B. Consequently, the vitellogenin gene (*MeVg1*) expression level was upregulated in a dose-dependent way. In this way, the maximum *MeVg1* transcript level in the hepatopancreas explants treated with 0.3 nm recombinant MeMIH-B was reached. Furthermore, an increase in the *MeVg1* expression in the hepatopancreas was detected when the shrimps were injected with recombinant MeMIH-B. Moreover, the vitellogenin-like immunoreactive protein showed a corresponding increase in the gonad and hemolymph of these female shrimps. Female shrimps were injected with MeMIH-B dsRNA and a significant reduction in the MeMIH-B transcript level in the thoracic ganglion and eyestalk was observed. There was also a significant drop of *MeVg1* expression in the hepatopancreas and ovary, and the vitellogenin level in the hemolymph was also reduced. This study demonstrated that the combined use of recombinant protein and RNAi tools can elucidate the function of MeMIH-B in vitellogenesis in *M. ensis*.

## 4. Ethical Issues and Future of Gene Editing

Ethically, the benefits must be greater than the risks. The use of the CRISPR-Cas9 technique can be risky and harmful since it may produce off-target mutations [153]. The process is complex, and it includes numerous ethical, bioethical and technical issues that can influence the performance of genome-editing technology. Such tools and technologies have resulted in the development of mutations that can cause several side effects when they are administered without the appropriate protocol [68]. This technique can also lead to cell death or alteration through the cutting of unintended sequences creating mutations [154]. To reduce the off-target mutations, efforts via new variants of the Cas9 enzyme have been made (eSpCas9, hypaCas9, Cas9HF-1), but these need further improvements, such as accurate modifications for therapeutic interference [155,156]. The CRISPR method was previously a technical ‘disruptor’, and we should contemplate how it can be turned into a ‘health disruptor’ [157,158]. The main disadvantage is the cost of the tools, techniques and reagents that are applied in the procedures [11]. In addition, there are risks to the environment. The unplanned release of the genetically modified (GM) experimental organisms into the natural world can cause gene drive extinction of the experimental population. Consequently, this leads to radical outcomes in the natural balance of the ecosystem [9,159]. Gene drive is a process of biased inheritance of genetic variants in a population in a non-Mendelian way [160]. The conversion efficiencies of the CRISPR-Cas9 editors of targeted gene drive mechanisms have been described as higher than 98% [161]. The off-target mutations can amplify in each generation, and it is risky to transfer genes and modified sequences to other species. The negative characteristics can be transmitted to related organisms far and wide. Therefore, the dispersion of the gene drive trait may be difficult to control [162]. Furthermore, it makes it more difficult to identify the GM organism outside the lab, thanks to precise genetic modifications through the effective CRISPR/Cas9 method. There are additional aspects that also play serious roles: the health effects of an allergic reaction to GM products and the environmental effects of the uncontrolled release of transgenes. Additionally, the diversity of natural genomes is reduced. This is demonstrated in the sociocultural aspect of “playing God” [163]. 

Genomic selection is on the threshold of becoming a reality and is making affected impressions in the genetic development of livestock. The betterment of the genes of aquatic species is a continuing process [164]. In the future, the best genotypes for aquaculture applications will be developed via traditional selective breeding together with new biotechnologies and molecular/genomic methods. To grow aquaculture production, more management tools will be required, especially in genetic enhancement, which has a strong potential to efficiently and sustainably enhance production. Genetic improvement can be revolutionizing with a non-transgenic method with highly effective gene editing tools. Genetic enhancement in aquatic creatures develops quickly, and the food production, competence and potential environmental impressions using genetic improvement appears promising for the future. Transgenic salmon were lately accepted for public consumption. If there is a public acceptance of transgenic fish flesh in the marketplace, then genetic enhancement of aquacultured organisms will dramatically increase [45]. However, the publicity could not tolerate the rapidly expanding CRISPR zoo. The regulation of patents and economic interests creates issues that are more challenging. Patents make it possible for biotechnological companies to have excessive power and benefits; on the other hand, patents will also support regulation in the field. In addition, through the practice of patenting, there are probably initiatives, litigation and friction between researchers and biotechnological companies. Thanks to the genome editing technology, there are several important advancements in biomedical research; however, it is presented with various challenges [9]. 

## 5. Conclusions

In modern times, the aquaculture industry is an essential sector of food production and global trade. On the grounds of the biological advantages of fish models, numerous novel protocols have accomplished gene modification in different fish species over the last few years. These studies demonstrate that gene editing tools, including the CRISPR/Cas9 technique, are very effective and widely used in aquaculture. It is applied in a broad range of fish species, extending from species with special adaptations (e.g., cavefish) to evolutionarily primitive species (e.g., lamprey), as well as from large species with economic relevance (e.g., Atlantic salmon) to model organisms (e.g., zebrafish) and cell lines (e.g., ZFL, SJD, ZF4). The targeted modifications in the genomic DNA of different fish species may bring radical changes in aquaculture production in the future. These make it possible to improve characteristics in aquaculture, like disease resistance, growth or reproduction. In addition, RNAi plays a crucial role in the silencing of gene expression. With this novel technique presenting an eco-friendly molecular device, the RNAi-mediated gene knockdown of a target gene has become possible. It can also influence the development of functional genomics and therapeutic applications in fish species and crustaceans. In summary, the creation of mutant animals in aquaculture through specific gene modification methods is the reality.

## Figures and Tables

**Table 1:** Comparison of three significant genome editing tools—ZFNs, TALENs and CRISPR [9].

Feature	Zinc Finger Nucleases (Zfns)	Transcription Activator-Like Effector Nucleases (Talens)	Clustered Regularly Interspaced Short Palindromic Repeats (CRISPR)
Structure	Fusion of zinc finger DNA-binding domain (DBD) with DNA-cleavage domain of Fok I endonuclease	Fusion of transcription activator-like effector DNA (TALEN) repeats with DNA-cleavage domain of Fok I endonuclease	Cas9 endonuclease and guide RNA (gRNA)
Size of recognition site	9–18 bases in DNA	30–36 bases in DNA	23 DNA bases in DNA
Ease of designing	More difficult than TALENs and CRISPR	Easier than ZFNs	Easier than the other two
Multiplexing	No	No	Yes
Off target	Same as that of TALENs	Same as that of ZFNs	More than the other two
Ease of redesigning/adaptability to target new site	Difficult, require recording of large DNA segments (500–1000 bp)		Easy, only requires a change in 20-bp protospacer of gRNA
Viral delivery	Using lentivirus and adenovirus; needs cotransduction with two lentiviral vectors, each encoding a monomer to form functional heterodimer	Using adenovirus	More difficult than the other two because it requires polyadenylation signal and promoter
Efficacy	+ +	+ +	+ + +
Application	Indels, obligate ligation-gated recombination (ObLiGaRe); can insert a 15-kb inducible gene expression cassette at a defined locus in human cell lines, tag ligation		Indels
Cost	Higher than CRISPR	Higher than CRISPR	Less than the other two

**Table 2:** Effective applications of the RNAi technique in fish [59].

Type of Molecule	Genes Targeted	RNAi	Response	Organism
		Specific	Nonspecific	
Long dsRNA	*ntl*, *fl h*, *pax2.1*, *LacZ*	x	x	Zebrafish embryos ^a^
Long dsRNA	*Gfp*, *Zf-T*, *pax6.1*	x		Zebrafish embryos
Long dsRNA	*Tbx16/spt*, *LacZ*		x	Zebrafish embryos
Long dsRNA	*pouII-1*, *gfp*, *terra*		x	Zebrafish embryos
Long dsRNA	*RanBP1*		x	Zebrafish embryos
Long dsRNA	*M2mAchR*	x		Zebrafish embryos
siRNA	*gfp*, *tyrA*	x		Rainbow trout embryos ^b^
siRNA	*Dmd*	x		Zebrafish embryos
esiRNA	*Ntl*		x	Zebrafish embryos
siRNA	*Ntl*	x		Zebrafish embryos
siRNA	*laminA* and *B2*, *Eg5*, *GL2*, *gfp*	x		ZFL, SJD and ZF4 ^c^
siRNA	*laminA*, *GL2*, *gfp*		x	Zebrafish embryos
Long dsRNA	*Myostatin*	x		Zebrafish embryos
T7RPshRNA	*ntl*, *gfp*	x		Zebrafish embryos

dsRNA: double-stranded RNA; Long dsRNA: double-stranded RNA > 30 nt; siRNA: small interfering RNA (21–25nt); esiRNA: endoribonuclease digestion-derived siRNA; T7RP-shRNA: short-hairpin RNA (shRNA) expression system, based on T7 RNA polymerase (T7RP)-directed transcription machinery. ^a^ *Danio rerio*. ^b^ *Oncorhynchus mykiss*. ^c^ Cell lines derived from adult and embryonic zebrafish (*Danio rerio*).

**Table 3:** Effective applications of the RNAi technique in crustaceans [59].

RNA	Target	Genes	RNAi Response	Organism
dsRNA	Endogenous ^A^	Spalt	Pleiotropic effects	*Artemia franciscana*
dsRNA		Chh	Decrease in glucose levels	*Litopenaeus schmitti*
dsRNA		ALF	Protection against WSSV	*Pacifastacus leniusculus*
dsRNA		pmYRP65	Inhibition of YHV cell entry	*Penaeus monodon* ^C^
dsRNA		proPO	Increased bacterial ^D^ growth	*Pacifastacus leniusculus*
dsRNA		Pacifastin	Decreased bacterial ^D^ growth	*Pacifastacus leniusculus*
dsRNA		Mih-B	Reduction of vitellogenin gene	*Metapenaeus ensis*
dsRNA		Pem-GIH	Decrease in *Pem-GIH* transcripts and reduction of vitellogenin gene	*Penaeus monodon*
dsRNA	Virals ^B^ and (unrelated)	hel, pol, pro, gp116, gp64	Inhibition of YHV replication	*Penaeus monodon* ^C^
dsRNA		(gfp)	Non-specific antiviral immunity	
dsRNA		vp28, vp15	Non-specific antiviral immunity and lower viral protection	*Penaeus monodon*
dsRNA		Pro	Inhibition of YHV replication	*Penaeus monodon*
dsRNA		(gfp, TSV pol)	Partial inhibition of YHV replication	
siRNA		(duck *u*)	Non-specific antiviral immunity	*Litopenaeus vannamei*
siRNA		vp28	Non-specific antiviral immunity	*Penaeus japonicus*

^A^ Produced sequence-specific response. ^B^ Produced both non-sequence-specific and sequence-specific antiviral immune reactions. ^C^ Primary culture of lymphoid ‘Oka’ cells. ^D^ *Aeromonas hydrophila.*

## Data Availability

Not applicable.

## Abbreviations

ZFN	Zinc finger nuclease
TALEN	Transcriptional activator-like effector nuclease
HDR	Homology-directed repair
NHEJ	Nonhomologous end joining
DSB	Double-strand break
CRISPR	Clustered regularly interspaced short palindromic repeats
sgRNA/gRNA	Single guide RNA
crRNA	CRISPR RNA
tracrRNA	Trans-activating crRNA
PAM	Protospacer adjacent motif
RNAi	RNA interference
dsRNA	Double-stranded RNA
siRNA	Small interfering RNA
hpRNA	Hairpin RNA
shRNA	Short-hairpin RNA
Ago	Argonaute protein
RISC	RNA-induced silencing complex
RdRp	RNA-dependent RNA polymerase
SVCV	Spring viraemia of carp virus
EPC	Epithelioma papulosum cyprinid
CyHV	3-cyprinid herpesvirus-3
TK	Thymidine kinase
DP	DNA polymerase
shRNA	Short hairpin RNA
MSTN	Myostatin
KO	Knockout
GM	Genetically modified
GH	Growth hormone
DHA	Docosahexaenoic acid
EPA	Eicosapentaenoic acid
CCB	Common carp brain
ALF	Anti-lipopolysaccharide factor
WSSV	White spot syndrome virus
CHH	Crustacean hyperglycemic hormone
PO	Phenoloxidase
proPO	Prophenoloxidase
SEM	Scanning electron microscopy
YHV	Yellow head virus
TSV	Taura syndrome virus
GIH	Gonad-inhibiting hormone
Vg	Vitellogenin
CHH	Crustacean hyperglycemic hormone
MIH	Molt-inhibiting hormone
GIH	Gonad-inhibiting hormone
eGFP	Exogenous GFP

## References

[1] Edwards P., Zhang W., Belton B., Little D.C. (2019). Misunderstandings, myths and mantras in aquaculture: Its contribution to world food supplies has been systematically over reported. Mar. Policy.

[2] FAO (2022). The State of World Fisheries and Aquaculture 2022.

[3] Food and Agriculture Organization (FAO) (2014). FAO: Fisheries and Aquaculture Statistics.

[4] FAO (2012). The State of World Fisheries and Aquaculture 2012.

[5] Boyd C.E., D’Abramo L.R., Glencross B.D., Huyben D.C., Juarez L.M., Lockwood G.S., McNevin A.A., Tacon A.G.J., Teletchea F., Tomasso J.R. (2020). Achieving sustainable aquaculture: Historical and current perspectives and future needs and challenges. J. World Aquac. Soc..

[6] World Bank (2013). Fish to 2030: Prospects for Fisheries and Aquaculture—World Bank Report Number 83177-GLB.

[7] Perota A., Lagutina I., Quadalti C., Lazzari G., Cozzi E., Galli C. (2016). The Applications of Genome Editing in Xenotransplantation. J. Genet. Genom..

[8] Cathomen T., Joung J.K. (2008). Zinc-finger Nucleases: The Next Generation Emerges. Mol. Ther..

[9] Malik Y.S., Barh D., Azevedo V.A.D.C., Khurana S.P. (2020). Genomics and Biotechnological Advances in Veterinary, Poultry, and Fisheries.

[10] Gratacap R.L., Wargelius A., Edvardsen R.B., Houston R.D. (2019). Potential of Genome Editing to Improve Aquaculture Breeding and Production. Trends Genet..

[11] Karre A. (2020). Gene Editing Technology. https://www.researchgate.net/publication/347442835_GENE_EDITING_TECHNOLOGY.

[12] Bibikova M., Beumer K., Trautman J.K., Carroll D. (2003). Enhancing Gene Targeting with Designed Zinc Finger Nucleases. Science.

[13] Moscou M.J., Bogdanove A.J. (2009). A Simple Cipher Governs DNA Recognition by TAL Effectors. Science.

[14] Egelie K.J., Graff G., Strand S.P., Johansen B. (2016). The emerging patent landscape of CRISPR–Cas gene editing technology. Nat. Biotechnol..

[15] Xu X., Hulshoff M.S., Tan X., Zeisberg M., Zeisberg E.M. (2020). CRISPR/Cas Derivatives as Novel Gene Modulating Tools: Possibilities and In Vivo Applications. Int. J. Mol. Sci..

[16] Takata M., Sasaki M.S., Sonoda E., Morrison C., Hashimoto M., Utsumi H., Yamaguchi-Iwai Y., Shinohara A., Takeda S. (1998). Homologous recombination and non-homologous end-joining pathways of DNA double-strand break repair have overlapping roles in the maintenance of chromosomal integrity in vertebrate cells. EMBO J..

[17] Barnes D.E. (2001). Non-homologous end joining as a mechanism of DNA repair. Curr. Biol..

[18] Lieber M.R. (2010). The Mechanism of Double-Strand DNA Break Repair by the Nonhomologous DNA End-Joining Pathway. Annu. Rev. Biochem..

[19] Lans H., Marteijn J.A., Vermeulen W. (2012). ATP-dependent chromatin remodeling in the DNA-damage response. Epigenetics Chromatin.

[20] Swiech L., Heidenreich M., Banerjee A., Habib N., Li Y., Trombetta J.J., Sur M., Zhang F. (2014). In vivo interrogation of gene function in the mammalian brain using CRISPR-Cas9. Nat. Biotechnol..

[21] Davis A.J., Chen D.J. (2013). DNA double strand break repair via non-homologous end-joining. Transl. Cancer Res..

[22] Chu V.T., Weber T., Wefers B., Wurst W., Sander S., Rajewsky K., Kühn R. (2015). Increasing the efficiency of homology-directed repair for CRISPR-Cas9-induced precise gene editing in mammalian cells. Nat. Biotechnol..

[23] Liu T., Huang J. (2016). DNA End Resection: Facts and Mechanisms. Genom. Proteom. Bioinform..

[24] Saleh-Gohari N., Helleday T. (2004). Conservative homologous recombination preferentially repairs DNA double-strand breaks in the S phase of the cell cycle in human cells. Nucleic Acids Res..

[25] Bharati J., Punetha M., Kumar B.S., Vidyalakshmi G., Sarkar M., D’Occhio M.J., Singh R.K. (2019). Genome editing in animals: An overview. Genomics and Biotechnological Advances in Veterinary, Poultry, and Fisheries.

[26] Choo Y., Sánchez-García I., Klug A. (1994). In vivo repression by a site-specific DNA-binding protein designed against an oncogenic sequence. Nature.

[27] Kim Y.G., Cha J., Chandrasegaran S. (1996). Hybrid restriction enzymes: Zinc finger fusions to Fok I cleavage domain. Proc. Natl. Acad. Sci. USA.

[28] Urnov F.D., Rebar E.J., Holmes M.C., Zhang H.S., Gregory P.D. (2010). Genome editing with engineered zinc finger nucleases. Nat. Rev. Genet..

[29] Tang L.-M., Zhou C.-L., Guo Z.-F., Xiao L., Chüeh A.C. (2015). Advances in Zinc Finger Nuclease and Its Applications. Gene Gene Ed..

[30] Carroll D. (2014). Genome Engineering with Targetable Nucleases. Annu. Rev. Biochem..

[31] MacPherson S., Larochelle M., Turcotte B. (2006). A Fungal Family of Transcriptional Regulators: The Zinc Cluster Proteins. Microbiol. Mol. Biol. Rev..

[32] Liu Q., Segal D.J., Ghiara J.B., Barbas C.F. (1997). Design of polydactyl zinc-finger proteins for unique addressing within complex genomes. Proc. Natl. Acad. Sci. USA.

[33] Palpant N.J., Dudzinski D.M. (2013). Zinc finger nucleases: Looking toward translation. Gene Ther..

[34] Meng X., Noyes M.B., Zhu L.J., Lawson N.D., Wolfe S.A. (2008). Targeted gene inactivation in zebrafish using engineered zinc-finger nucleases. Nat. Biotechnol..

[35] Urnov F.D., Miller J.C., Lee Y.-L., Beausejour C.M., Rock J.M., Augustus S., Jamieson A.C., Porteus M.H., Gregory P.D., Holmes M.C. (2005). Highly efficient endogenous human gene correction using designed zinc-finger nucleases. Nature.

[36] Hockemeyer D., Soldner F., Beard C., Gao Q., Mitalipova M., DeKelver R.C., Katibah G.E., Amora R., Boydston E.A., Zeitler B. (2009). Efficient targeting of expressed and silent genes in human ESCs and iPSCs using zinc-finger nucleases. Nat. Biotechnol..

[37] Joung J.K., Sander J.D. (2013). TALENs: A widely applicable technology for targeted genome editing. Nat. Rev. Mol. Cell Biol..

[38] Boch J., Scholze H., Schornack S., Landgraf A., Hahn S., Kay S., Lahaye T., Nickstadt A., Bonas U. (2009). Breaking the Code of DNA Binding Specificity of TAL-Type III Effectors. Science.

[39] Boch J., Bonas U. (2010). *Xanthomonas* AvrBs3 Family-Type III Effectors: Discovery and Function. Annu. Rev. Phytopathol..

[40] Miller J.C., Tan S., Qiao G., Barlow K.A., Wang J., Xia D.F., Meng X., Paschon D.E., Leung E., Hinkley S.J. (2011). A TALE nuclease architecture for efficient genome editing. Nat. Biotechnol..

[41] Lamb B.M., Mercer A.C., Barbas C.F. (2013). Directed evolution of the TALE N-terminal domain for recognition of all 5′ bases. Nucleic Acids Res..

[42] Mahfouz M.M., Li L., Shamimuzzaman, Wibowo A., Fang X., Zhu J.-K. (2011). De novo-engineered transcription activator-like effector (TALE) hybrid nuclease with novel DNA binding specificity creates double-strand breaks. Proc. Natl. Acad. Sci. USA.

[43] Jinek M., Chylinski K., Fonfara I., Hauer M., Doudna J.A., Charpentier E. (2012). A Programmable dual-RNA-guided DNA endonuclease in adaptive bacterial immunity. Science.

[44] Crudele J.M., Chamberlain J.S. (2018). Cas9 immunity creates challenges for CRISPR gene editing therapies. Nat. Commun..

[45] Lucas J.S., Southgate P.C., Tucker C.S. (2013). Aquaculture: Farming Aquatic Animals and Plants.

[46] Pankaj C.Y. (2014). Cell Stem Cell. Sci Rep..

[47] Mali P., Esvelt K.M., Church G. (2013). Cas9 as a versatile tool for engineering biology. Nat. Methods.

[48] Grissa I., Vergnaud G., Pourcel C. (2007). CRISPRFinder: A web tool to identify clustered regularly interspaced short palindromic repeats. Nucleic Acids Res..

[49] Brouns S.J.J., Jore M.M., Lundgren M., Westra E.R., Slijkhuis R.J.H., Snijders A.P.L., Dickman M.J., Makarova K.S., Koonin E.V., Van Der Oost J. (2008). Small CRISPR RNAs Guide Antiviral Defense in Prokaryotes. Science.

[50] Makarova K.S., Haft D.H., Barrangou R., Brouns S.J.J., Charpentier E., Horvath P., Moineau S., Mojica F.J.M., Wolf Y.I., Yakunin A.F. (2011). Evolution and classification of the CRISPR–Cas systems. Nat. Rev. Microbiol..

[51] Wiedenheft B., Lander G.C., Zhou K., Jore M.M., Brouns S.J.J., Van Der Oost J., Doudna J.A., Nogales E. (2011). Structures of the RNA-guided surveillance complex from a bacterial immune system. Nature.

[52] Li M., Wang D. (2017). Gene editing nuclease and its application in tilapia. Sci. Bull..

[53] Jinek M., Jiang F., Taylor D.W., Sternberg S.H., Kaya E., Ma E., Anders C., Hauer M., Zhou K., Lin S. (2014). Structures of Cas9 endonukleases reveal RNA-mediated conformational activation. Science.

[54] Gersbach C.A. (2014). Genome engineering: The next genomic revolution. Nat. Methods.

[55] Lees-Miller S.P., Meek K. (2003). Repair of DNA double strand breaks by non-homologous end joining. Biochimie.

[56] Singh I.P., Hasan S., Saxena P. (2019). RNAi: Gene Silencing. Int. J. Res. Eng. Sci. Manag..

[57] Hood E. (2004). RNAi: What’s all the noise about gene silencing?. Environ. Health Perspect..

[58] Daneholt B. (2006). Advanced Information on the Nobel Prize in Physiology or Medicine 2006.

[59] Estrada M.P., Lugo J.M., Carpio Y. (2008). RNAi in fish and crustaceans. RNA Interference: Methods for Plants and Animals.

[60] Sen G.L., Blau H.M. (2006). A brief history of RNAi: The silence of the genes. FASEB J..

[61] Singh D., Chaudhary S., Kumar R., Sirohi P., Mehla K., Sirohi A., Kumar S., Chand P., Singh P.K. (2016). RNA Interference Technology—Applications and Limitations. RNA Interference.

[62] Borges F., Martienssen R.A. (2015). The expanding world of small RNAs in plants. Nat. Rev. Mol. Cell Biol..

[63] Majumdar R., Rajasekaran K., Cary J.W. (2017). RNA Interference (RNAi) as a Potential Tool for Control of Mycotoxin Contamination in Crop Plants: Concepts and Considerations. Front. Plant Sci..

[64] Parveen P., Brundavani K.D., Mahathi K., Bhavani M.S., Shaheda Sultana S.K. (2019). Gene Silencing and DNA Methylation. Am. J. Phytomedicine Clin. Therapeutics..

[65] Walsh A.S., Yin H., Erben C.M., Wood M.J.A., Turberfield A.J. (2011). DNA Cage Delivery to Mammalian Cells. ACS Nano.

[66] Keum J.-W., Ahn J.-H., Bermudez H. (2011). Design, Assembly, and Activity of Antisense DNA Nanostructures. Small.

[67] Tirasophon W., Roshorm Y., Panyim S. (2005). Silencing of yellow head virus replication in penaeid shrimp cells by dsRNA. Biochem. Biophys. Res. Commun..

[68] Tiu S.H.-K., Chan S.-M. (2007). The use of recombinant protein and RNA interference approaches to study the reproductive functions of a gonad-stimulating hormone from the shrimp Metapenaeus ensis. FEBS J..

[69] Naito Y., Yamada T., Ui-Tei K., Morishita S., Saigo K. (2004). siDirect: Highly effective, target-specific siRNA design software for mammalian RNA interference. Nucleic Acids Res..

[70] Hannon G.J. (2002). RNA interference. Nature.

[71] Wang N., Sun Y.-H., Liu J., Wu G., Su J.-G., Wang Y.-P., Zhu Z.-Y. (2007). Knock down of gfp and no tail expression in zebrafish embryo by in vivo-transcribed short hairpin RNA with T7 plasmid system. J. Biomed. Sci..

[72] Gotesman M., Menanteau-Ledouble S., Saleh M., Bergmann S.M., El-Matbouli M. (2018). A new age in AquaMedicine: Unconventional approach in studying aquatic diseases. BMC Vet. Res..

[73] Okoli A.S., Blix T., Myhr A.I., Xu W., Xu X. (2021). Sustainable use of CRISPR/Cas in fish aquaculture: The biosafety perspective. Transgenic Res..

[74] Lieschke G.J., Currie P.D. (2007). Animal models of human disease: Zebrafish swim into view. Nat. Rev. Genet..

[75] Wang F., Shi Z., Cui Y., Guo X., Shi Y.-B., Chen Y. (2015). Targeted gene disruption in Xenopus laevis using CRISPR/Cas9. Cell Biosci..

[76] Doyon Y., McCammon J.M., Miller J.C., Faraji F., Ngo C., Katibah G.E., Amora R., Hocking T.D., Zhang L., Rebar E.J. (2008). Heritable targeted gene disruption in zebrafish using designed zinc-finger nucleases. Nat. Biotechnol..

[77] Auer T.O., Duroure K., De Cian A., Concordet J.-P., Del Bene F. (2014). Highly efficient CRISPR/Cas9-mediated knock-in in zebrafish by homology-independent DNA repair. Genome Res..

[78] Edvardsen R., Leininger S., Kleppe L., Skaftnesmo K.O., Wargelius A. (2014). Targeted Mutagenesis in Atlantic Salmon (*Salmo salar* L.) Using the CRISPR/Cas9 System Induces Complete Knockout Individuals in the F0 Generation. PLoS ONE.

[79] Qiu C., Cheng B., Zhang Y., Huang R., Liao L., Li Y., Luo D., Hu W., Wang Y. (2014). Efficient Knockout of Transplanted Green Fluorescent Protein Gene in Medaka Using TALENs. Mar. Biotechnol..

[80] Li M., Yang H., Zhao J., Fang L., Shi H., Li M., Sun Y., Zhang X., Jiang D., Zhou L. (2014). Efficient and Heritable Gene Targeting in Tilapia by CRISPR/Cas9. Genetics.

[81] Matsuda M., Nagahama Y., Shinomiya A., Sato T., Matsuda C., Kobayashi T., Morrey C.E., Shibata N., Asakawa S., Shimizu N. (2002). DMY is a Y-specific DM-domain gene required for male development in the medaka fish. Nature.

[82] Karigo T., Aikawa M., Kondo C., Abe H., Kanda S., Oka Y. (2014). Whole Brain-Pituitary In Vitro Preparation of the Transgenic Medaka (Oryzias latipes) as a Tool for Analyzing the Differential Regulatory Mechanisms of LH and FSH Release. Endocrinology.

[83] Karigo T., Kanda S., Takahashi A., Abe H., Okubo K., Oka Y. (2012). Time-of-Day-Dependent Changes in GnRH1 Neuronal Activities and Gonadotropin mRNA Expression in a Daily Spawning Fish, Medaka. Endocrinology.

[84] Cattanach B.M., Iddon C.A., Charlton H.M., Chiappa S.A., Fink G. (1977). Gonadotrophin-releasing hormone deficiency in a mutant mouse with hypogonadism. Nature.

[85] Takahashi A., Kanda S., Abe T., Oka Y. (2016). Evolution of the Hypothalamic-Pituitary-Gonadal Axis Regulation in Vertebrates Revealed by Knockout Medaka. Endocrinology.

[86] Qin Z., Li Y., Su B., Cheng Q., Ye Z., Perera D.A., Fobes M., Shang M., Dunham R.A. (2016). Editing of the Luteinizing Hormone Gene to Sterilize Channel Catfish, Ictalurus punctatus, Using a Modified Zinc Finger Nuclease Technology with Electroporation. Mar. Biotechnol..

[87] Taranger G.L., Karlsen Ø., Bannister R.J., Glover K.A., Husa V., Karlsbakk E., Kvamme B.O., Boxaspen K.K., Bjørn P.A., Finstad B. (2015). Risk assessment of the environmental impact of Norwegian Atlantic salmon farming. ICES J. Mar. Sci..

[88] Glover K.A., Quintela M., Wennevik V., Besnier F., Sørvik A.G.E., Skaala Ø. (2012). Three Decades of Farmed Escapees in the Wild: A Spatio-Temporal Analysis of Atlantic Salmon Population Genetic Structure throughout Norway. PLoS ONE.

[89] Wargelius A., Leininger S., Skaftnesmo K.O., Kleppe L., Andersson E., Taranger G.L., Schulz R.W., Edvardsen R.B. (2016). Dnd knockout ablates germ cells and demonstrates germ cell independent sex differentiation in Atlantic salmon. Sci. Rep..

[90] Youngren K.K., Coveney D., Peng X., Bhattacharya C., Schmidt L.S., Nickerson M.L., Lamb B.T., Deng J.M., Behringer R.R., Capel B. (2005). The Ter mutation in the dead end gene causes germ cell loss and testicular germ cell tumours. Nature.

[91] Dungan H.M., Clifton D.K., Steiner R.A. (2006). Minireview: Kisspeptin Neurons as Central Processors in the Regulation of Gonadotropin-Releasing Hormone Secretion. Endocrinology.

[92] Lee D.K., Nguyen T., O’Neill G.P., Cheng R., Liu Y., Howard A.D., Coulombe N., Tan C.P., Tang-Nguyen A.-T., George S.R. (1999). Discovery of a receptor related to the galanin receptors. FEBS Lett..

[93] Popa S.M., Clifton D.K., Steiner R.A. (2008). The Role of Kisspeptins and GPR54 in the Neuroendocrine Regulation of Reproduction. Annu. Rev. Physiol..

[94] Oakley A.E., Clifton D.K., Steiner R.A. (2009). Kisspeptin Signaling in the Brain. Endocr. Rev..

[95] Roa J., Aguilar E., Dieguez C., Pinilla L., Tena-Sempere M. (2008). New frontiers in kisspeptin/GPR54 physiology as fundamental gatekeepers of reproductive function. Front. Neuroendocr..

[96] Tang H., Liu Y., Luo D., Ogawa S., Yin Y., Li S., Zhang Y., Hu W., Parhar I.S., Lin H. (2015). The kiss/kissr Systems Are Dispensable for Zebrafish Reproduction: Evidence From Gene Knockout Studies. Endocrinology.

[97] Zhong Z., Niu P., Wang M., Huang G., Xu S., Sun Y., Xu X., Hou Y., Sun X., Yan Y. (2016). Targeted disruption of sp7 and myostatin with CRISPR-Cas9 results in severe bone defects and more muscular cells in common carp. Sci. Rep..

[98] Dahlem T.J., Hoshijima K., Jurynec M.J., Gunther D., Starker C., Locke A.S., Weis A., Voytas D., Grunwald D.J. (2012). Simple Methods for Generating and Detecting Locus-Specific Mutations Induced with TALENs in the Zebrafish Genome. PLOS Genet..

[99] Jao L.-E., Wente S.R., Chen W. (2013). Efficient multiplex biallelic zebrafish genome editing using a CRISPR nuclease system. Proc. Natl. Acad. Sci. USA.

[100] Ma L., Jeffery W.R., Essner J.J., Kowalko J.E. (2015). Genome Editing Using TALENs in Blind Mexican Cavefish, Astyanax mexicanus. PLoS ONE.

[101] Blackburn P.R., Campbell J.M., Clark K.J., Ekker S.C. (2013). The CRISPR System—Keeping Zebrafish Gene Targeting Fresh. Zebrafish.

[102] Segev-Hadar A., Slosman T., Rozen A., Sherman A., Cnaani A., Biran J. (2021). Genome Editing Using the CRISPR-Cas9 System to Generate a Solid-Red Germline of Nile Tilapia (*Oreochromis niloticus*). CRISPR J..

[103] Souza T.A., Chen X., Guo Y., Sava P., Zhang J., Hill J.J., Yaworsky P.J., Qiu Y. (2008). Proteomic Identification and Functional Validation of Activins and Bone Morphogenetic Protein 11 as Candidate Novel Muscle Mass Regulators. Mol. Endocrinol..

[104] McPherron A.C., Lawler A.M., Lee S.J. (1997). Regulation of skeletal muscle mass in mice by a new TGF-beta superfamily member. Nature.

[105] Lee S.-J., McPherron A.C. (2001). Regulation of myostatin activity and muscle growth. Proc. Natl. Acad. Sci. USA.

[106] Khalil K., Elayat M., Khalifa E., Daghash S., Elaswad A., Miller M., Abdelrahman H., Ye Z., Odin R., Drescher D. (2017). Generation of Myostatin Gene-Edited Channel Catfish (Ictalurus punctatus) via Zygote Injection of CRISPR/Cas9 System. Sci. Rep..

[107] Lilley J.H., Callinan R.B., Chinabut S., Kanchanakhan S., MacRae I.H. (1998). Epizootic Ulcerative Syndrome (EUS) Technical Handbook.

[108] (2017). OIE (World Organisation for Animal Health) Listed Diseases. https://www.oie.int/en/what-we-do/animal-health-and-welfare/animal-diseases/.

[109] Yike I. (2011). Fungal Proteases and Their Pathophysiological Effects. Mycopathologia.

[110] Majeed M., Kumar G., Schlosser S., El-Matbouli M., Saleh M. (2017). In vitro investigations on extracellular proteins secreted by Aphanomyces invadans, the causative agent of epizootic ulcerative syndrome. Acta Vet. Scand..

[111] Majeed M., Soliman H., Kumar G., El-Matbouli M., Saleh M. (2018). Editing the genome of Aphanomyces invadans using CRISPR/Cas9. Parasites Vectors.

[112] Roberts R.J., Willoughby L.G., Chinabut S. (1993). Mycotic aspects of epizootic ulcerative syndrome (EUS) of Asian fishes. J. Fish Dis..

[113] Pathiratne A., Widanapathirana G.S., Chandrakanthi W.H.S. (1994). Association of Aeromonas hydrophila with epizootic ulcerative syndrome (EUS) of freshwater fish in Sri Lanka. J. Appl. Ichthyol..

[114] Lilley J.H., Roberts R.J. (1997). Pathogenicity and culture studies comparing the Aphanomyces involved in epizootic ulcerative syndrome (EUS) with other similar fungi. J. Fish Dis..

[115] Vishwanath T., Mohan C., Shankar K. (1998). Epizootic Ulcerative Syndrome (EUS), associated with a fungal pathogen, in Indian fishes: Histopathology—‘A cause for invasiveness’. Aquaculture.

[116] Biacchesi S. (2011). The reverse genetics applied to fish RNA viruses. Vet. Res..

[117] Reshi M.L., Wu J.-L., Wang H.-V., Hong J.-R. (2014). RNA interference technology used for the study of aquatic virus infections. Fish Shellfish. Immunol..

[118] Lima P.C., Harris J.O., Cook M. (2013). Exploring RNAi as a therapeutic strategy for controlling disease in aquaculture. Fish Shellfish. Immunol..

[119] Carpio Y., Estrada M.P. (2006). Zebrafish as a genetic model organism. Biotecnol. Apl..

[120] Hammond S.M. (2006). MicroRNA therapeutics: A new niche for antisense nucleic acids. Trends Mol. Med..

[121] Gotesman M., Soliman H., Besch R., El-Matbouli M. (2015). Inhibition of spring viraemia of carp virus replication in an *E pithelioma papulosum cyprini* cell line by RNA i. J. Fish Dis..

[122] Gotesman M., Soliman H., Besch R., El-Matbouli M. (2014). In vitro inhibition of Cyprinid herpesvirus-3 replication by RNAi. J. Virol. Methods.

[123] Adamek M., Rauch G., Brogden G., Steinhagen D. (2014). Small interfering RNA treatment can inhibit Cyprinid herpesvirus 3 associated cell death in vitro. Pol. J. Vet. Sci..

[124] Saleh M., Kumar G., Abdel-Baki A.-A., Dkhil M.A., El-Matbouli M., Al-Quraishy S. (2016). In Vitro Gene Silencing of the Fish Microsporidian *Heterosporis saurida* by RNA Interference. Nucleic Acid Ther..

[125] Hofer B. (1903). Über die Drehkrankheit der Regenbogenforelle. Allg Fisch-Ztg..

[126] Sarker S., El-Matbouli M. (2015). Can RNAi Target Salmonid Whirling Disease In Vivo?. Nucleic Acid Ther..

[127] Sarker S., Kallert D., Hedrick R., El-Matbouli M. (2015). Whirling disease revisited: Pathogenesis, parasite biology and disease intervention. Dis. Aquat. Org..

[128] Sarker S., Menanteau-Ledouble S., Kotob M.H., El-Matbouli M. (2017). A RNAi-based therapeutic proof of concept targets salmonid whirling disease in vivo. PLoS ONE.

[129] De Rienzo G., Gutzman J.H., Sive H., Don E.K., Formella I., Badrock A.P., Hall T.E., Morsch M., Hortle E., Hogan A. (2012). Efficient shRNA-Mediated Inhibition of Gene Expression in Zebrafish. Zebrafish.

[130] Bartel D.P. (2009). MicroRNAs: Target Recognition and Regulatory Functions. Cell.

[131] Gruber J., Manninga H., Tuschl T., Osborn M., Weber K. (2005). Specific RNAi Mediated Gene Knockdown in Zebrafish Cell Lines. RNA Biol..

[132] Saraiva M., de Bruijn I., Grenville-Briggs L., McLaggan D., Willems A., Bulone V., van West P. (2014). Functional characterization of a tyrosinase gene from the oomycete Saprolegnia parasitica by RNAi silencing. Fungal Biol..

[133] Capodici J., Karikó K., Weissman D. (2002). Inhibition of HIV-1 Infection by Small Interfering RNA-Mediated RNA Interference. J. Immunol..

[134] Fire A., Xu S., Montgomery M.K., Kostas S.A., Driver S.E., Mello C.C. (1998). Potent and specific genetic interference by double-stranded RNA in Caenorhabditis elegans. Nature.

[135] Winston W.M., Molodowitch C., Hunter C.P. (2002). Systemic RNAi in *C. elegans* Requires the Putative Transmembrane Protein SID-1. Science.

[136] Feinberg E.H., Hunter C.P. (2003). Transport of dsRNA into Cells by the Transmembrane Protein SID-1. Science.

[137] Grishok A. (2005). RNAi mechanisms in *Caenorhabditis elegans*. FEBS Lett..

[138] Flegel T. (1997). Major viral diseases of the black tiger prawn (Penaeus monodon) in Thailand. World J. Microbiol. Biotechnol..

[139] Lightner D.V., Hasson K.W., White B.L., Redman R.M. (1998). Experimental infection of western hemisphere penaeid shrimp with asian white spot syndrome virus and asian yellow head virus. J. Aquat. Anim. Health.

[140] Assavalapsakul W., Smith D.R., Panyim S. (2006). Identification and Characterization of a *Penaeus monodon* Lymphoid Cell-Expressed Receptor for the Yellow Head Virus. J. Virol..

[141] Westenberg M., Heinhuis B., Zuidema D., Vlak J.M. (2005). siRNA injection induces sequence-independent protection in Penaeus monodon against white spot syndrome virus. Virus Res..

[142] Witteveldt J., Vermeesch A.M.G., Langenhof M., de Lang A., Vlak J.M., van Hulten M.C.W. (2005). Nucleocapsid protein VP15 is the basic DNA binding protein of white spot syndrome virus of shrimp. Arch. Virol..

[143] Van Hulten M.C., Witteveldt J., Snippe M., Vlak J.M. (2001). White Spot Syndrome Virus Envelope Protein VP28 Is Involved in the Systemic Infection of Shrimp. Virology.

[144] Liu H., Jiravanichpaisal P., Söderhäll I., Cerenius L., Söderhäll K. (2006). Antilipopolysaccharide Factor Interferes with White Spot Syndrome Virus Replication In Vitro and In Vivo in the Crayfish *Pacifastacus leniusculus*. J. Virol..

[145] Liu H., Jiravanichpaisal P., Cerenius L., Lee B.L., Söderhäll I., Söderhäll K. (2007). Phenoloxidase Is an Important Component of the Defense against Aeromonas hydrophila Infection in a Crustacean, Pacifastacus leniusculus. J. Biol. Chem..

[146] Soyez D., Noel P., Van Deijnen J., Martin M., Morel A., Payen G. (1990). Neuropeptides from the sinus gland of the lobster Homarus americanus: Characterization of hyperglycemic peptides. Gen. Comp. Endocrinol..

[147] Lugo J.M., Morera Y., Rodríguez T., Huberman A., Ramos L., Estrada M.P. (2006). Molecular cloning and characterization of the crustacean hyperglycemic hormone cDNA from *Litopenaeus schmitti*: Functional analysis by double-stranded RNA interference technique. FEBS J..

[148] Copf T., Rabet N., Averof M. (2006). Knockdown of spalt function by RNAi causes de-repression of Hox genes and homeotic transformations in the crustacean Artemia franciscana. Dev. Biol..

[149] Treerattrakool S., Panyim S., Chan S.-M., Withyachumnarnkul B., Udomkit A. (2008). Molecular characterization of gonad-inhibiting hormone of Penaeus monodon and elucidation of its inhibitory role in vitellogenin expression by RNA interference. FEBS J..

[150] Keller R. (1992). Crustacean neuropeptides: Structures, functions and comparative aspects. Experientia.

[151] De Kleijn D.P., Van Herp F. (1995). Molecular biology of neurohormone precursors in the eyestalk of Crustacea. Comp. Biochem. Physiol. Part B Biochem. Mol. Biol..

[152] Chan S.-M., Gu P.-L., Chu K.H., Tobe S.S. (2003). Crustacean neuropeptide genes of the CHH/MIH/GIH family: Implications from molecular studies. Gen. Comp. Endocrinol..

[153] Yang L., Güell M., Byrne S., Yang J.L., Angeles A.D.L., Mali P., Aach J., Kim-Kiselak C., Briggs A.W., Rios X. (2013). Optimization of scarless human stem cell genome editing. Nucleic Acids Res..

[154] Fu Y., Foden J.A., Khayter C., Maeder M.L., Reyon D., Joung J.K., Sander J.D. (2013). High-frequency off-target mutagenesis induced by CRISPR-Cas nucleases in human cells. Nat. Biotechnol..

[155] Cong L., Ran F.A., Cox D., Lin S., Barretto R., Habib N., Hsu P.D., Wu X., Jiang W., Marraffini L.A. (2013). Multiplex Genome Engineering Using CRISPR/Cas Systems. Science.

[156] Hsu P.D., Scott D.A., Weinstein J.A., Ran F.A., Konermann S., Agarwala V., Li Y., Fine E.J., Wu X., Shalem O. (2013). DNA targeting specificity of RNA-guided Cas9 nucleases. Nat. Biotechnol..

[157] Ledford H. (2015). CRISPR, the disruptor. Nature.

[158] Capps B., Chadwick R., Joly Y., Mulvihill J.J., Lysaght T., Zwart H. (2017). Falling giants and the rise of gene editing: Ethics, private interests and the public good. Hum. Genom..

[159] Oye K.A., Esvelt K., Appleton E., Catteruccia F., Church G., Kuiken T., Lightfoot S.B.-Y., McNamara J., Smidler A., Collins J.P. (2014). Regulating gene drives. Science.

[160] Collins J.P. (2018). Gene drives in our future: Challenges of and opportunities for using a self-sustaining technology in pest and vector management. BMC Proc..

[161] Gantz V.M., Bier E. (2015). The mutagenic chain reaction: A method for converting heterozygous to homozygous mutations. Science.

[162] Esvelt K.M., Smidler A.L., Catteruccia F., Church G.M. (2014). Concerning RNA-guided gene drives for the alteration of wild populations. eLife.

[163] Hackett P.B., Fahrenkrug S.C., Carlson D.F. (2014). The Promises and Challenges of Precision Gene Editing in Animals of Agricultural Importance.

[164] Zhu B., Ge W. (2018). Genome editing in fishes and their applications. Gen. Comp. Endocrinol..

